# Dynamic behaviour of interphases and its implication on high-energy-density cathode materials in lithium-ion batteries

**DOI:** 10.1038/ncomms14589

**Published:** 2017-04-26

**Authors:** Wangda Li, Andrei Dolocan, Pilgun Oh, Hugo Celio, Suhyeon Park, Jaephil Cho, Arumugam Manthiram

**Affiliations:** 1 Materials Science and Engineering Program and Texas Materials Institute, the University of Texas at Austin, Austin, Texas 78712, USA; 2 Department of Energy Engineering, School of Energy and Chemical Engineering, Ulsan National Institute of Science and Technology (UNIST), 689-798 Ulsan, South Korea

## Abstract

Undesired electrode–electrolyte interactions prevent the use of many high-energy-density cathode materials in practical lithium-ion batteries. Efforts to address their limited service life have predominantly focused on the active electrode materials and electrolytes. Here an advanced three-dimensional chemical and imaging analysis on a model material, the nickel-rich layered lithium transition-metal oxide, reveals the dynamic behaviour of cathode interphases driven by conductive carbon additives (carbon black) in a common nonaqueous electrolyte. Region-of-interest sensitive secondary-ion mass spectrometry shows that a cathode-electrolyte interphase, initially formed on carbon black with no electrochemical bias applied, readily passivates the cathode particles through mutual exchange of surface species. By tuning the interphase thickness, we demonstrate its robustness in suppressing the deterioration of the electrode/electrolyte interface during high-voltage cell operation. Our results provide insights on the formation and evolution of cathode interphases, facilitating development of *in situ* surface protection on high-energy-density cathode materials in lithium-based batteries.

## Abstract (web summary)

In lithium-ion batteries the interactions between the electrode and electrolyte represent a complex but critical process. Here the authors reveal the dynamic behaviour of interphases driven by conductive carbon through chemical and imaging analyses of a model transition-metal oxide cathode material.

Considerable interest has been directed towards the development of reliable, low-cost, high-energy-density rechargeable Li-ion batteries to meet the demands of multiple emerging fields, such as advanced robotics, electric vehicles and grid storage^1^,^2^,^3^,^4^,^5^. This has motivated extensive investigation of cathode materials with higher operating voltages than conventional LiCoO_2_ (upper cutoff voltage of ∼4.2 V vs Li/Li^+^) to maximize energy-storage capabilities. Potential candidates include Ni-rich layered oxides^6^,^7^,^8^ (LiNi_1-*x*_M_*x*_O_2_ with M=metal ∼4.5 V), Li-rich layered oxides^9^,^10^,^11^ (Li_1+*x*_M_1-*x*_O_2_ with ∼4.7 V), high-voltage spinel oxides^12^,^13^ (LiNi_0.5_Mn_1.5_O_4_ with ∼4.8 V) and high-voltage polyanionic compounds^14^,^15^,^16^ (phosphates and sulfates, up to ∼5.2 V). Unfortunately, these cathodes suffer from limited service life due to aggressive electrochemical degradation initiated at the electrode–electrolyte interface. This is largely caused by their operating voltages extending over the electrochemical stability window of most known nonaqueous electrolytes in lithium-ion batteries^2^,^4^,^5^,^17^. For instance, commercial ethylene carbonate (EC)-based electrolytes with lithium hexafluorophosphate (LiPF_6_) as the salt have a stability window of ∼1.3–4.3 V vs Li/Li^+^. Moreover, oxidative decomposition of electrolyte components at high-voltage operation is not the only concern; often, surface degradation on cathodes involves active mass dissolution (for example, Mn^2+^) (refs 13, 18, 19, 20) and irreversible structural reconstruction (for example, cation migration) at highly delithiated states^10^,^11^,^19^,^21^,^22^,^23^.

As the quest for alternative, non-reacting electrolyte combinations is a formidable task, most efforts tackling the unsatisfactory cycle life of these high-voltage cathodes attempt to enhance the electrode–electrolyte interface stability with the formation of a stable, robust passivation film, similar to the solid-electrolyte interphase formed on graphite^2^,^4^,^17^,^24^. This is achieved *ex situ* by coating or doping of active cathode particles^25^,^26^,^27^, or *in situ* by employing electrolyte additives^28^,^29^. Despite some modest success, effective passivation of the cathode material surface (up to ∼5 V vs Li/Li^+^) remains challenging in the battery community^2^,^4^,^5^. The understanding of interphases formed on cathode materials in existing cell configurations is rather limited as well, in part due to complex influences of the ‘inactive' conductive carbon additives (for example, carbon black) in composite electrodes, which have not been recognized until recently^14^,^17^,^26^,^30^,^31^,^32^. This is not surprising since commonly used surface-sensitive diagnostic tools, such as X-ray and infrared spectroscopies, cannot spatially separate different components in electrodes. Undoubtedly, studies on the dynamic formation and evolution of interphases (both spontaneous and under electrochemical cycling) involving the active material and carbon altogether are of pivotal importance and should provide insights on the disputable effects of cathode interphases^4^,^5^,^14^,^18^,^33^,^34^,^35^ on electrochemical properties and for further optimization.

Here we use time-of-flight secondary-ion mass spectrometry (TOF-SIMS) to investigate the electrode–electrolyte interphases and their impacts on overall cell performance on a Ni-rich layered oxide cathode (LiNi_0.7_Co_0.15_Mn_0.15_O_2_), with a particular focus on the role of conductive carbon. TOF-SIMS offers sub-nanometer surface sensitivity and ultra-high chemical selectivity, and more importantly, region-of-interest (ROI) analysis can be applied to the composite electrode, enabling individual components to be observed independently^36^. Our results reveal the dominant influence of carbon on the surface chemistry of composite cathode electrodes in common nonaqueous electrolytes. The carbon-driven interphase formed *in situ* on LiNi_0.7_Co_0.15_Mn_0.15_O_2_ before cell operation serves as a passivating film against deleterious interfacial reactions with the electrolyte.

## Results

### Visualization of interphases on cycled LiNi_0.7_Co_0.15_Mn_0.15_O_2_

**Figure 1:**
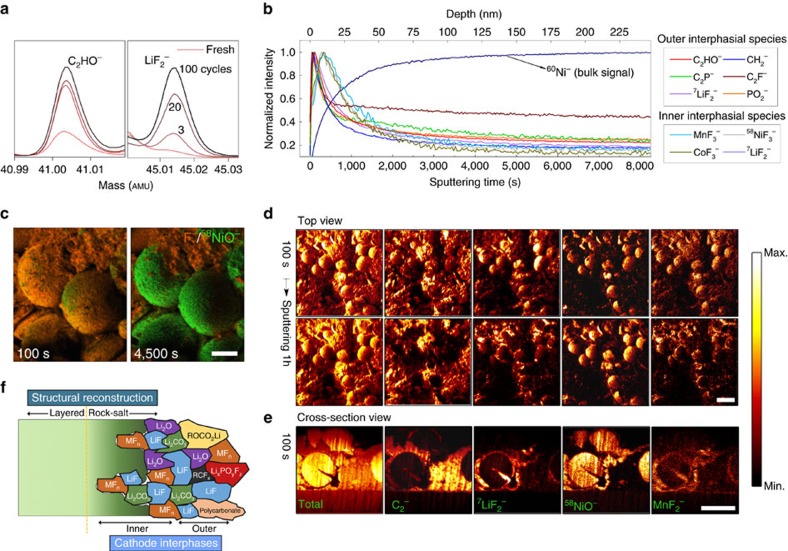
Localization and visualization of interphases at the surface of cycled LiNi_0.7_Mn_0.15_Co_0.15_O_2_. (**a**) TOF-SIMS spectra of C_2_HO^−^ and ^7^LiF_2_^−^ integrated over 1,000 s of Cs^+^ sputtering (10 s sampling step) on LiNi_0.7_Mn_0.15_Co_0.15_O_2_ composite electrodes after battery operation at room temperature, representative of the evolution of organic and inorganic interphasial species as a function of cycles. (**b**) Normalized (to maximum) depth profiling of several secondary ion fragments of interest at the cycled LiNi_0.7_Mn_0.15_Co_0.15_O_2_ surface. Depth is calculated based on the calibrated Cs^+^ sputtering rate of ∼0.03 nm s^−1^ for the active material (Supplementary Fig. 1). (**c**–**e**) TOF-SIMS chemical mapping (burst alignment mode) on cycled LiNi_0.7_Mn_0.15_Co_0.15_O_2_ electrodes. (**c**) Illustrative maps of F^−^ and ^58^NiO^−^ before and after intensive Cs^+^ sputtering, depicting the interphases on the composite electrode of secondary particles, additive carbon and polymeric binder (100 s shallow milling was applied in order to reduce possible contamination, such as adventitious chemisorbed carbon). Scale bar, 10 μm. (**d**,**e**) Comparative chemical mapping of several fragments, unraveling the complex interphases on the electrode. (**d**) Shows top views collected after 100 s and 1 h Cs^+^ etching, while **e** is taken from the cross-sectional perspective. From the left to right, the secondary ions of interest are, respectively, ‘total', C_2_^−^, ^7^LiF_2_^−^, ^58^NiO^−^ and MnF_2_^−^. Scale bars, 20 μm in both **d**,**e**. (**f**) A schematic diagram of the microstructure and chemical composition of surface degradation products at the of surface LiNi_0.7_Mn_0.15_Co_0.15_O_2_. The size of regions is not drawn to scale.

Figure 1a,b illustrate, respectively, the TOF-SIMS spectra and depth profiles of secondary ions of interest on LiNi_0.7_Co_0.15_Mn_0.15_O_2_ electrodes during high-voltage electrochemical operation. In Fig. 1a, we witness rapid generation and subsequent marginal, prolonged buildup of organic interphasial species (represented by C_2_HO^−^), together with continuous accumulation of inorganic species during cycling (^7^LiF_2_^−^); the depth profiles in Fig. 1b, on the other hand, reveal the complex multi-layer characteristics of the interphases on LiNi_1-*x*_M_*x*_O_2_ particles. Both findings here are consistent with literature results^5^,^13^,^17^,^18^,^33^, where overall thickening of cathode interphases during cycling is often observed^4^,^34^,^37^. It has been noted that degradation products, including LiF and many organic species, appear localized at the very surface of the interphases (the outer layer), while transition-metal fluorides show high concentrations in the inner layer (where LiF is also abundant). These two ‘layers' correspond to multiple processes of interfacial evolution of LiNi_1-*x*_M_*x*_O_2_ in common LiPF_6_/EC-based solutions: (i) deposition of salt and solvent (oxidative) decomposition products from the electrolyte^4^,^5^,^18^,^33^,^35^ and (ii) active mass dissolution aggravated by acidic species attack^5^,^18^,^25^,^33^ (for example, HF—generated in the presence of trace electrolyte impurities, such as H_2_O). Of particular note, another essential process, the intrinsic structural reconstruction from the layered to the ‘rock-salt' phase (NiO), also occurs when destabilized transition-metal ions migrate towards neighbouring vacant Li sites at highly delithiated states^19^,^21^,^22^.

Illustrative TOF-SIMS chemical mappings were collected on LiNi_0.7_Co_0.15_Mn_0.15_O_2_ composite electrodes after cycling (Fig. 1c). These maps show that the surface of active cathode particles (represented by the ^58^NiO^−^ fragment), initially covered entirely by degradation-induced chemical species (represented by F^−^), emerges upon Cs^+^ sputtering. Detailed visualization in three dimensions of the chemical alterations occurring on the same sample is shown in Fig. 1d,e, and Supplementary Figs 2–4. Additional mappings on the pristine electrodes can be found in Supplementary Fig. 5. The typical surface degradation products in the outer and inner interphases^4^,^5^,^18^,^33^,^35^ are evidenced by cumulative signals of C_2_^−^, ^7^LiF_2_^−^ and MnF_2_^−^ in the top view before and after intensive Cs^+^ etching in Fig. 1d, as well as in the cross-sectional view with shallow Cs^+^ milling in Fig. 1e. These degradation products appear mostly located at the surface of secondary particles, although electrolyte penetration along with further attack through micro-cracks towards their interior is possible^6^,^38^,^39^. Notably, transition-metal species (^58^NiO^−^ and MnF_2_^−^) are found on the carbon black/binder surface. It has been shown that the dissolution products from cathode particles eventually make their ways to the graphite anode surface while migrating within the electrolyte during cell operation^4^,^7^,^12^,^13^,^34^. A schematic diagram of the complex electrode-electrolyte interfacial chemical and structural evolution during battery operation is depicted in Fig. 1f, with further details in Supplementary Table 2.

### Spontaneous interphase formation on composite cathode electrodes

**Figure 2:**
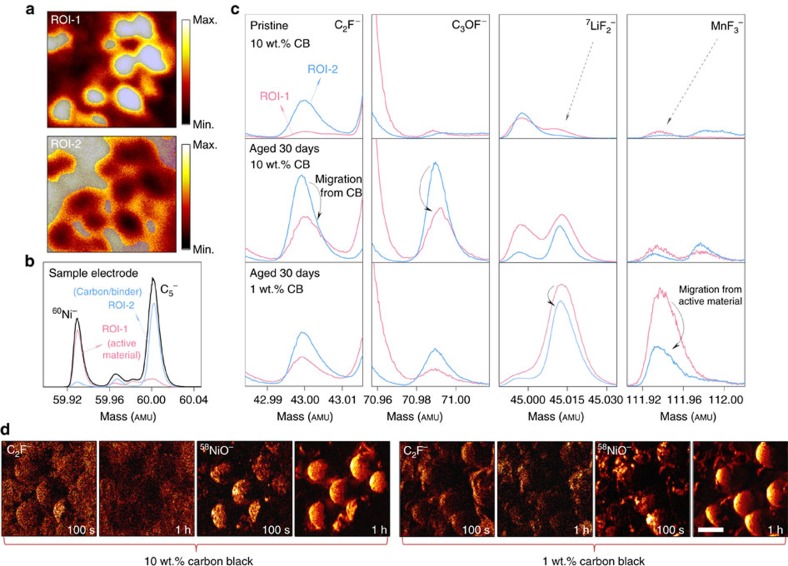
Spontaneous formation of interphases on LiNi_0.7_Mn_0.15_Co_0.15_O_2_ composite electrodes during aging. (**a**) TOF-SIMS chemical maps of two secondary ion fragments, ^58^NiO^−^ (upper) and C_3_H^−^ (lower), on a sample LiNi_0.7_Mn_0.15_Co_0.15_O_2_ electrode. ROI-1 is applied to retrieve signals from the active material region (represented by ^58^NiO^−^) while ROI-2 is used for the carbon/binder area (C_3_H^−^), respectively. (**b**) Illustrative TOF-SIMS spectra of ^60^Ni^−^ and C_5_^−^ on the same sample electrode, demonstrating the effectiveness of ROI selection in spatially separating different components in the composite electrode. (**c**) TOF-SIMS spectra collected on pristine (10 wt.% carbon black, shortened for ‘CB' in the figure), 30-day aged (1 and 10 wt.% carbon black) electrodes, with ROI-1 and ROI-2 applied, respectively. The data are integrated over 600 s of sputtering time with two scans per 10 s. Several fragments of interests are, respectively, C_2_F^−^, C_3_OF^−^, ^7^LiF_2_^−^ and MnF_3_^−^ from left to right, along with CH_2_^−^, C_3_H_2_^−^, C_2_P^−^, C_3_O_2_F^−^, MnF_2_^−^, C_5_OF^−^, ^58^NiF_3_^−^ and CoF_3_^−^ in Supplementary Figs 6 and 7. All spectra are normalized by ROI coverage and drawn to the same scale in each panel. Note that C_2_F^−^ can also be produced by the binder (polyvinylidene fluoride), hence the signal counts observed on the pristine electrode (ROI-2). (**d**) TOF-SIMS high-resolution mapping (burst alignment mode) of C_2_F^−^ and ^58^NiO^−^ on the electrodes aged for 30 days before and after 1 h of Cs^+^ sputtering (100 s shallow milling was applied to reduced adventitious contamination). It can be seen that the cathode particles undergo severe dissolution/fragmentation (represented by ^58^NiO^−^ signals) in the electrode containing 1 wt.% carbon black, while for the 10 wt.% one, the CEI (C_2_F^−^) species show a larger amount on the particles. Scale bar, 20 μm.

Motivated by some recent studies^14^,^26^,^30^,^31^,^32^ reporting the dominating influence of additive conductive carbon (carbon black) on the behaviour of cathode interphases, we carried out TOF-SIMS measurements aided by ROI selection on aged LiNi_0.7_Co_0.15_Mn_0.15_O_2_ composite electrodes in LiPF_6_/EC-DEC. Figure 2a demonstrates TOF-SIMS chemical mapping of two secondary ion fragments, ^58^NiO^−^ (upper) and C_3_H^−^ (lower), at the electrode surface, representative of active cathode particles and carbon black/binder, respectively. Two ROIs (ROI-1 and ROI-2) are used accordingly to extract the spectra in Fig. 2b,c. Clearly, the ROI selections are highly efficient in spatially separating the fragments of interest, ^60^Ni^−^ (ROI-1) and C_5_^−^ (ROI-2), as seen in Fig. 2b. In Fig. 2c, a series of TOF-SIMS spectra of C_2_F^−^, C_3_OF^−^, ^7^LiF_2_^−^ and MnF_3_^−^, were retrieved from pristine and aged electrodes for 30 days with different amounts of carbon black (1 and 10 wt.%). Readily obvious is the increase of all four species after aging in both ROI-1 and ROI-2, indicative of ongoing electrode–electrolyte interactions before any electrical bias applied. The migration of active mass dissolution products (represented by ^7^LiF_2_^−^ and MnF_3_^−^, as well as MnF_2_^−^, ^58^NiF_3_^−^ and CoF_3_^−^ in Supplementary Fig. 6) from the active particles towards carbon/binder regions is also evident, as these signals increase simultaneously in ROI-2. Interestingly, a dramatically larger amount of the dissolution products is found at the aged electrode surface with 1 wt.% carbon black compared with that with 10 wt.%. Moreover, in Fig. 2d, maps of ^58^NiO^−^ on these aged electrodes suggest a much more severe extent of particle dissolution for the 1 wt.% carbon electrode. This indicates that the active cathode particles could be passivated against acid leaching from the electrolyte with the involvement of carbon black during interphases formation. Further evidence is needed, however, since carbon black, with a much larger relative surface area compared with LiNi_0.7_Co_0.15_Mn_0.15_O_2_, serves as an effective acidic species scavenger for the 10 wt.% carbon electrode.

The surface species responsible for the C_2_F^−^ and C_3_OF^−^ fragments observed here originate primarily from two processes: (i) direct acidic species attack (HF) at the surface of carbon black, where dangling bonds of carbon and surface chemical functional groups (such as hydroxyl and carbonyl groups) are abundant and particularly susceptible^26^,^30^ and (ii) acidic species attack on carbonate solvents in LiPF_6_/EC-DEC electrolyte. In Fig. 2c, we notice that (i) in the ROI-1 spectra, intensities of C_2_F^−^ and C_3_OF^−^ are consistently lower than those in ROI-2, and (ii) the relative amounts of these fragments in ROI-1 compared with the pristine electrodes are increasing with that of carbon black. Additional evidence from fragments such as C_3_H_2_^−^, C_3_O_2_F^−^ and C_5_OF^−^ in good agreement can be found in Supplementary Fig. 7. These findings strongly support that the spontaneously produced surface deposits during aging, such as fluorinated alkane and carbonate species, are mainly a result of acidic species attack on carbon and actively migrate to the cathode particles surface once formed. The resulting surface layer is hereafter referred to as a cathode-electrolyte interphase (CEI), which further evolves upon cell operation. Note that the CEI defined in the current study only refers to the interphasial species produced from spontaneous reactions between carbon black and LiPF_6_/EC-DEC. Chemical mapping provides further support of such migration (Fig. 2d), with the cathode particles of 10 wt.% carbon black evidently covered by C_2_F^−^ species; whereas for the 1 wt.% electrode, the coverage is less complete. Considering the well-known migration of dissolution products from cathode particles shown above, there exists a dynamic ‘communication' between the active cathode material and conductive carbon additives during the spontaneous interphases formation in Li-ion batteries, which should have a profound impact on the electrochemical properties.

### Tuning the thickness of spontaneously formed CEI

**Figure 3:**
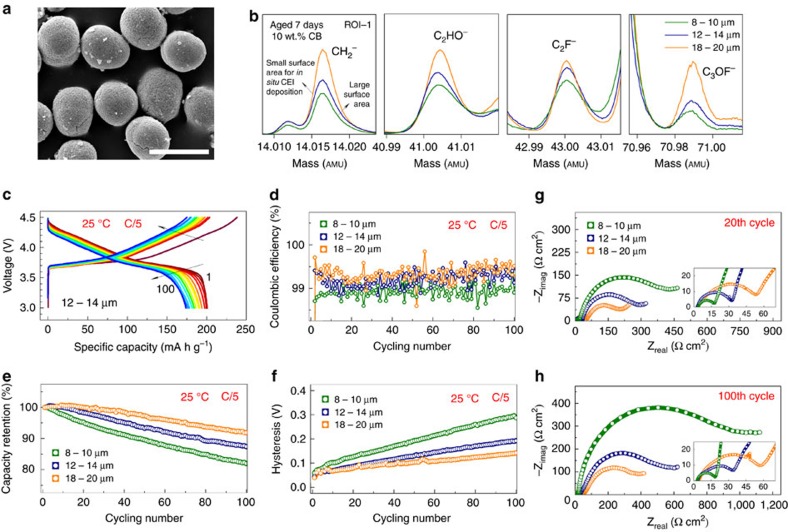
Morphology and electrochemical characterizations of LiNi_0.7_Mn_0.15_Co_0.15_O_2_. (**a**) SEM image of the as-prepared LiNi_0.7_Mn_0.15_Co_0.15_O_2_ powder of 12–14 μm in size. Samples prepared through the same method with different secondary particle sizes (8–10 and 18–20 μm) are shown in Supplementary Fig. 8. The specific surface area of the three samples was determined to be, respectively, 0.60, 0.53, and 0.46 m^2^ g^−1^ via the Brunauer-Emmett-Teller method (Supplementary Table 1). Scale bar, 20 μm. (**b**) TOF-SIMS spectra of several CEI species (ROI-1 applied and normalized by ROI coverage; integrated over 1,000 s of Cs^+^ sputtering, 10 s interval, two scans per step) on aged LiNi_0.7_Mn_0.15_Co_0.15_O_2_ particles after 7 days. Additional spectra of CH_3_O^−^, C_3_H_2_^−^, ^7^LiF_2_^−^, C_3_O_2_F^−^, C_5_OF^−^, MnF_3_^−^, ^58^NiF_3_^−^ and CoF_3_^−^ can be found in Supplementary Fig. 10; the protection of CEI on the active material against acid leaching is readily noticeable among the three samples. (**c**–**f**) Galvanostatic charge-discharge tests of three LiNi_0.7_Mn_0.15_Co_0.15_O_2_ electrodes of different particle size at room temperature. (**c**) The evolution of charge-discharge profiles during 100 cycles at C/5 for the 12–14 μm sample, (**d**,**e**,**f**) comparison of their Coulombic efficiency, normalized (to maximum) cycling stability and hysteresis (defined here as the difference between average charge and discharge voltages) as cycling proceeds. 1C is equal to 180 mA g^−1^. The electrochemical profiles of LiNi_1-*x*_M_*x*_O_2_ materials are characterized by a gradually sloping line with no indication of irreversible oxygen release or bulk layered-to-‘spinel-like' phase transition, commonly seen for Li-rich layered Li_1+*x*_M_1-*x*_O_2_ (refs 9, 10). (**g**,**h**) Nyquist plots showing the impedance evolution of three LiNi_0.7_Mn_0.15_Co_0.15_O_2_ discharged composite electrodes of different particle sizes upon cycling (to 3.0 V vs Li/Li^+^): (**g**) 20 cycles, and (**h**) 100 cycles. The inset shows the magnified semicircle at the high-frequency region in corresponding main figures. After refreshing with a new Li metal anode and electrolyte after 50 cycles, the cell performance and impedance (Supplementary Fig. 17) do not show improvement, confirming the deterioration of the cathode–electrolyte interface being the main reason for the sluggish charge-transfer kinetic properties; meanwhile, a Bode plot of three discharged electrodes after 100 cycles in Supplementary Fig. 18 indicates the interphases on Li anodes contribute to the first high-frequency semicircle.

To verify the passivation effects of CEI during high-voltage electrochemical reactions, we prepared three LiNi_0.7_Co_0.15_Mn_0.15_O_2_ samples with the same bulk chemical composition but different secondary particle sizes (Fig. 3a and Supplementary Fig. 8) through a transition-metal co-precipitation process^8^,^40^. This enables the direct modification of the CEI thickness, as the active material that has smaller relative surface area (larger particles, Supplementary Table 1) undergoes more *in situ* spontaneous deposition of surface species from carbon, as demonstrated in Fig. 3b. Powder X-ray diffraction (XRD) confirmed that all as-synthesized samples have a well-defined R
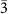
m layered structure with the cation mixing of around 3%, that is, the content of Ni ions in the Li layer (Supplementary Fig. 9)^19^,^22^,^41^. The electrochemical performance of LiNi_0.7_Co_0.15_Mn_0.15_O_2_ materials in lithium half cells was recorded at room temperature. Figure 3c exhibits typical charge-discharge profiles of LiNi_1-*x*_M_*x*_O_2_ with a cutoff voltage of 4.5 V vs Li/Li^+^ (12–14 μm particle size shown; 8–10 and 18–20 μm particle size in Supplementary Fig. 11). In Fig. 3d–f, the three samples demonstrate different Coulombic efficiencies, hysteresis and capacity fades as cycling proceeds; the sample with larger particles shows better cycling stability. As seen in secondary electron microscopy images of cycled electrodes in Supplementary Figs 12–14, the majority of LiNi_0.7_Co_0.15_Mn_0.15_O_2_ particles do not yet exhibit major mechanical fracture at such an early cycling stage (100 cycles) in contrast to many anodes, as their volume change during Li de-intercalation is small (∼5%). XRD on cycled electrodes (Supplementary Fig. 15) indicates a marginal increase in cation mixing while all samples retained a good overall layered structure with no apparent bulk phase transformation. Hence, the performance decline is primarily caused by rapid degradation of the electrode/electrolyte interface that induces large over-potentials during charge-discharge processes^6^,^7^,^19^,^21^,^22^,^23^, which is crucially related to the passivation effect of CEI.

Impedance spectroscopy was, therefore, employed to offer insight to the interfacial evolution for the three samples during cycling. Through this technique, the complex resistance or impedance of all components in an electrochemical cell can be determined^33^. Impedance spectra of LiNi_0.7_Co_0.15_Mn_0.15_O_2_/Li half cells at different cycles are given in Fig. 3g,h, and in Supplementary Fig. 16. As a rule, the lower intercept at very high frequencies represents combined influences of electrolyte, current collector and separator resistances. The impedances obtained here are mostly indexed to Li-ion migration through surface films formed (spontaneously and electrochemically) on both Li metal anodes and LiNi_0.7_Co_0.15_Mn_0.15_O_2_ cathodes (that is, the first high-frequency flat semicircle) and charge-transfer across the cathode active-material/surface-film interface (that is, the second semicircle at medium-to-low frequency range). Often, the interphasial species, such as ROCO_2_Li (refs 5, 33) and LiF (ref. 42), are highly resistive and yet enable facile Li diffusion during the intercalation process, while the interfacial charge transfer dominates and becomes greatly hurdled by the combined impacts of active mass dissolution and structural reconstruction at the particle surface. Indeed, the first semicircles are almost invisible compared with the second ones in the figure, indicative of negligible adverse kinetic impacts of CEI on Li-ion insertion/extraction. For each cell, the Li-ion transport appears more rapid for the sample of smaller particles, which can be ascribed to less CEI formation on the sample with larger surface area (Fig. 3b); meanwhile, a larger downward trend in the charge-transfer kinetics is observed. This explains the difference in cell performance among three samples in Fig. 3d–f and suggests that the CEI leads to suppression of the electrode/electrolyte interface deterioration during high-voltage battery operation.

### Passivation of the electrode/electrolyte interface by CEI

**Figure 4:**
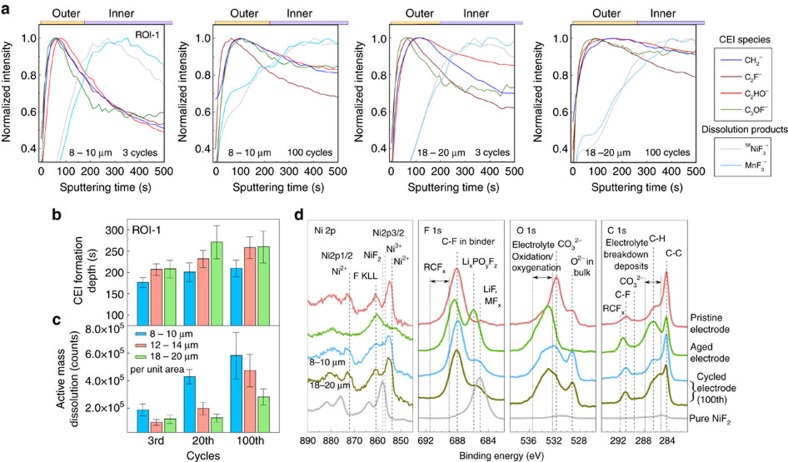
Quantification of surface degradation due to electrode-electrolyte reactivity. (**a**–**c**) TOF-SIMS characterizations on cycled LiNi_0.7_Mn_0.15_Co_0.15_O_2_ electrodes (ROI-1 applied) of different secondary particle sizes upon battery operation at room temperature. (**a**) Normalized (to maximum) depth profiles of CEI and active mass dissolution products fragments for electrodes of 8–10 and 18–20 μm after 3 and 100 cycles, respectively, with Cs^+^ sputtering. (**b**) comparison of the CEI formation depth for electrodes of different particle size and cycles. The formation depth was calculated based on the averaged peak positions of four representative CEI signal fragments depth profiles collected at multiple positions on the electrode. The error bar is defined by the s.d. of the peak positions for each fragment (for more details, refer to Supplementary Tables 3–5). (**c**) Comparison of the amount of dissolution products generated per unit area for electrodes of different particle sizes and cycles, demonstrating the variation of acidic species attack during cycling. This is obtained by integrating the spectra of MnF_3_^−^, ^58^NiF_3_^−^ and CoF_3_^−^ (ROI-1 applied, normalized by ROI coverage) over 750 s of Cs^+^ sputtering (10 s sampling step), as shown in Supplementary Tables 6–8. The error bar is defined by the s.d. of integrated intensities for each fragment. (**d**) XPS spectroscopic data of LiNi_0.7_Mn_0.15_Co_0.15_O_2_ electrodes and pure NiF_2_: Ni 2p, F 1s, O 1s and C 1s. Spectra of the pristine, aged and cycled electrodes (8–10 and 18–20 μm particle sizes) as well as NiF_2_ are displayed from the top to bottom. A more thorough series of XPS spectra for all samples can be found in Supplementary Figs 25 and 26. Note that the spontaneously generated surface film on the composite electrode upon exposure to the LiPF_6_/EC-DEC solution after aging is so thick that the bulk signals (features in O 1s and Ni 2p regions) completely vanish.

We carried out detailed surface chemical and structural characterizations to provide direct evidence of this passivation effect. Compared with the electrode aged for 30 days without any electrical bias applied, the cycled electrode over the same period of time exhibits less amount of CEI species at the surface; in the meantime, high-voltage cycling notably accelerates active mass dissolution (Supplementary Fig. 19). It is expected that the spontaneously formed CEI will partially decompose at the highly oxidizing environment of ∼4.5 V. In Fig. 4, we present a quantitative investigation of the surface degradation owing to electrode-electrolyte reactivity during cell operation. Comparison of normalized TOF-SIMS depth profiles of representative CEI signals with ROI-1 (active particles) taken on samples at various cycles (3, 20 and 100) are shown in Fig. 4a and in Supplementary Figs 20–24. Their corresponding calculated CEI formation depth as a function of cycles is illustrated in Fig. 4b. In Fig. 4a,b, we witness a clear correlation between the LiNi_0.7_Co_0.15_Mn_0.15_O_2_ secondary particle sizes and CEI formation depth (that is, larger particles lead to thicker CEIs). In contrast, such tendencies are reversed for the active mass dissolution products generated per unit area of the active material, which are more severe for the sample with smaller particles (Fig. 4c). Moreover, though damaged initially, the CEI appears somewhat stabilized during repeated charge-discharge cycles, whereas the dissolution products accumulate drastically. X-ray photoelectron spectroscopy (XPS) analysis in Fig. 4d reveals the interphases on cycled LiNi_0.7_Co_0.15_Mn_0.15_O_2_ electrodes similar to those reported in the literature on layered transition-metal oxides^9^,^18^,^23^,^30^ and further corroborates the differing degree of surface degradation among electrodes of different secondary particle sizes. The Ni 2p, F 1s, O 1s and C 1s regions high-resolution XPS spectra of representative pristine, aged and cycled electrodes, as well as pure NiF_2_, are depicted. The O 1s region reveals a native film mostly consisting of Li_2_CO_3_ on the LiNi_1-*x*_M_*x*_O_2_ pristine particles, and various electrolyte degradation products^18^,^24^ are generated during aging, such as semicarbonates, LiF, MF_*x*_, Li_*x*_PO_*y*_F_*z*_ and RCF_*x*_. In this study, as mentioned above, we focus on certain interphasial species that are mainly produced from spontaneous reactions between carbon black and LiPF_6_/EC-DEC (defined as the CEI here) and active mass dissolution products instead of those from other origins. For cycled electrodes, reduction in the amount of CEI species is noticed compared with the aged one (Fig. 4d). In support of TOF-SIMS results, the degree of dissolution products generation (NiF_2_) at the electrode surface is found larger for the sample with smaller secondary particles (Ni 2p, 8–10 μm); meanwhile, the CEI formation appears more pronounced for the sample with larger particle size (C 1s and O 1s, 18–20 μm). However, such comparison should be treated with caution, since XPS cannot selectively retrieve data from the interphases on the active material.

**Figure 5:**
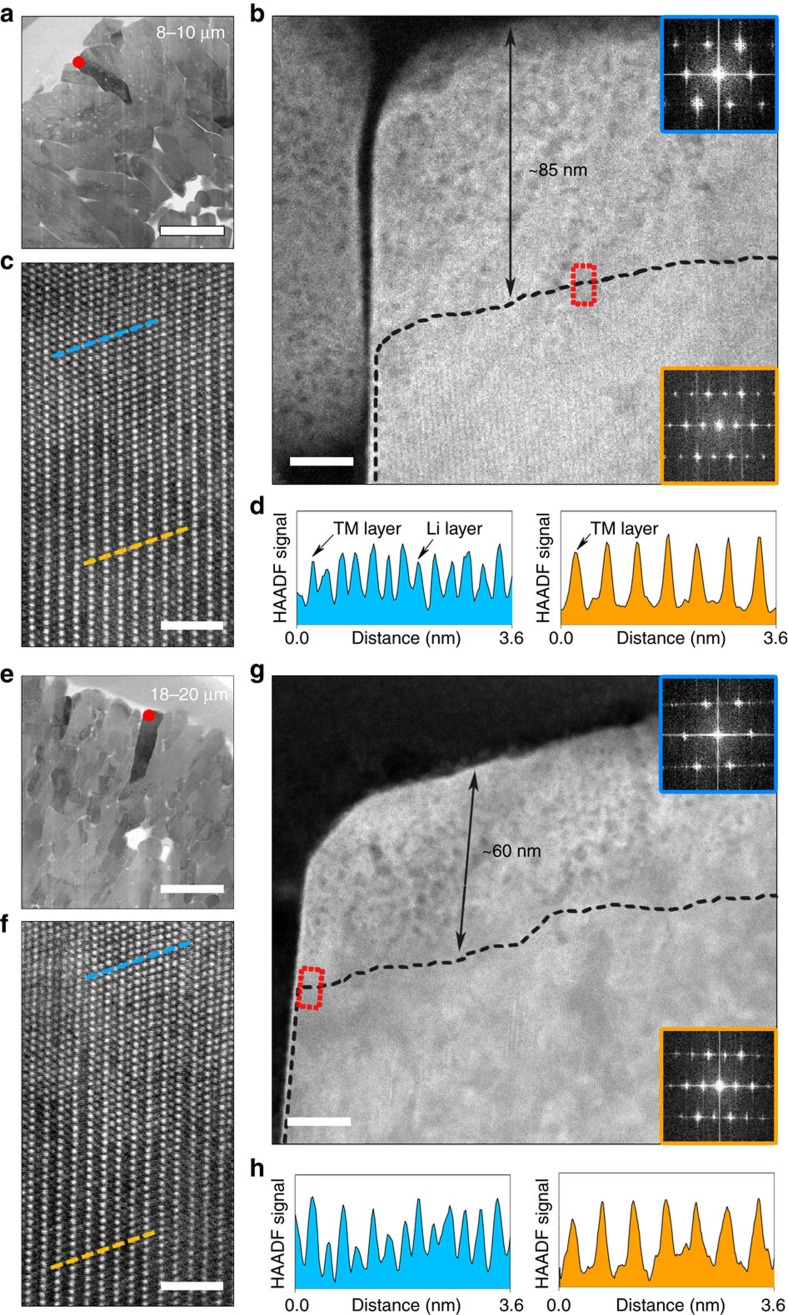
Quantification of surface degradation due to structural reconstruction. (**a**–**h**) Images of LiNi_0.7_Mn_0.15_Co_0.15_O_2_ particles surface after 100 cycles at room temperature): (**a**–**d**) 8–10 μm and (**e**–**h**) 18–20 μm. (**a**,**e**) High-resolution transmission electron micrographs illustrating the secondary particle morphology. (**b**,**f**) Magnified corresponding locations at a primary particle surface marked by a red dot in **a**,**e** with insets of fast Fourier transform patterns of regions divided by a dashed line. These patterns show the rock-salt phase, that is, Fm
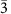
m [110] zone axis (in blue), and the pristine layered phase, R
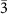
m [100] zone axis (in orange), respectively. (**c**,**g**) HAADF-STEM images of areas enclosed by a red rectangle in **b**,**f** indicating the local atomic arrangement along the interface region. (**d**,**h**) Corresponding HAADF signal profiles of the blue and orange dashed lines in **c**,**g** providing additional evidence of the different structures neighbouring the interface region. The scale bars in **a** and **e**, **b** and **g**, **c** and **f** are, respectively, 1 μm, 20 nm and 2 nm. Note that the CEI is largely invisible in these images owing to the destructive effect of high-energy electron-beam irradiation during STEM characterization^46^.

Another essential aspect of surface deterioration, that is, formation of the surface rock-salt phase (NiO) owing to intrinsic structure reconstruction, was also quantitatively analysed for LiNi_0.7_Co_0.15_Mn_0.15_O_2_ electrodes cycled under similar conditions. In Fig. 5, high-angle annular dark-field scanning transmission electron microscopy (HAADF-STEM) images are displayed for samples of 8–10 and 18–20 μm in secondary particles size. The two distinct regions of the rock-salt Fm
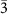
m and the original layered R
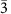
m structures, divided by a dashed line in Fig. 5b,g, are confirmed by corresponding fast Fourier transform patterns (Fig. 5b,g insets) and high-resolution HAADF images/signal-profiles taken at the interface region^19^,^21^,^22^ (Fig. 5c,d,f,h). As seen, the rock-salt phase becomes significantly thick after 100 cycles along lithium slabs (where Li-ion diffusion is more rapid) for both samples, and is featured by a porous structure from a clear difference in contrast between the outer surface and the inner bulk areas. This suggests that the highly localized active mass dissolution products form in corrosion pits, and are largely overlapped with the rock-salt phase regime at the surface. Note that it is generally challenging to spatially resolve these dissolved transition-metal species in high-resolution STEM characterization. HAADF-STEM patterns of pristine samples free of the electrochemically induced rock-salt phase can be found in Supplementary Figs 27–29. As seen, the extent of rock-salt phase formation for the two samples shows a similar tendency to that of dissolution products (Fig. 4c). TOF-SIMS depth profiles of referenced ratios between ^58^NiO^−^ and ^58^Ni^−^ (Supplementary Fig. 30), as well as XRD data for the three cycled electrodes^−^(Supplementary Fig. 15)—both of which can indicate the NiO formation—are in line with the HAADF-STEM results as well.

## Discussion

**Figure 6:**
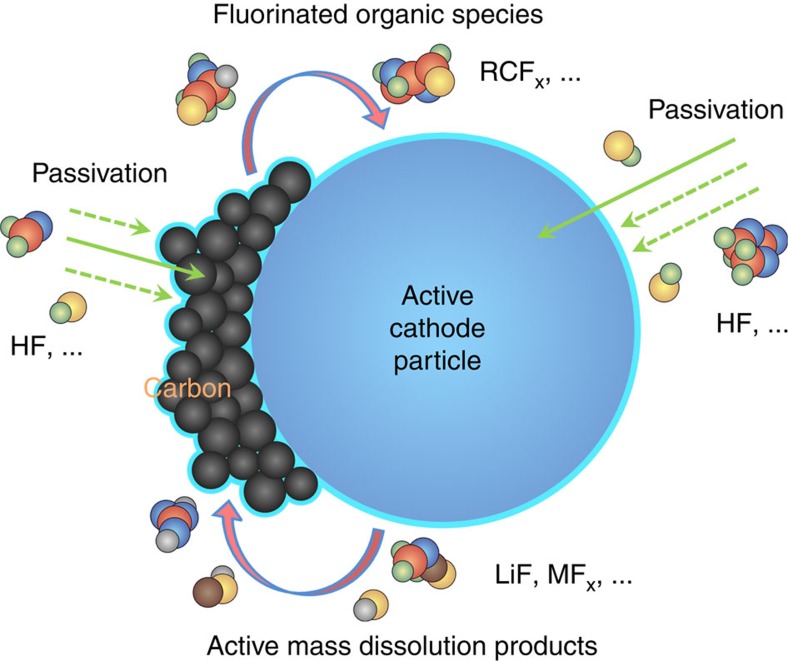
The dynamic behaviour of cathode interphases in Li-ion batteries. Schematic illustrating the evolution of surface chemistry on an active cathode particle (LiNi_0.7_Mn_0.15_Co_0.15_O_2_) and conductive carbon in composite electrodes in a simple LiPF_6_/EC-based electrolyte. The spontaneously formed CEI functions as a passivating film that suppresses electrode-electrolyte side reactions under electrochemical cycling, though with limited effectiveness. The species shown are not intended to represent specific compounds (other than HF) during the process and the sizes of the various components are not drawn to scale.

On the basis of above results, we conclude that before electrochemical operation, conductive carbon additives (carbon black) readily react with the LiPF_6_/EC-based electrolyte upon contact, giving rise to a CEI that further evolves during cell operation and passivates the LiNi_0.7_Co_0.15_Mn_0.15_O_2_ particles. By applying ROI analysis on the TOF-SIMS spectra, we show that this *in situ* generated CEI originates from the mutual exchange of interphasial species between the active cathode particles and carbon (Fig. 6). Undesired electrode–electrolyte interactions upon battery operation, however, cannot be entirely excluded due to incomplete CEI coverage, which also becomes further compromised in strongly oxidizing conditions up to 4.5 V vs Li/Li^+^. Moreover, we provide direct evidence of the overall protective nature of the CEI at cathode particles surface upon cycling, via TOF-SIMS, XPS and HAADF-STEM. By employing samples of different secondary particle sizes, we are able to alter the CEI thickness. For a fixed amount of carbon in the electrodes (10%), a thicker CEI adheres onto the active material with a smaller relative surface area (larger particles) during aging. Meanwhile, an unequivocal correlation between the thickness of CEI and the generation of degradation products is revealed among the three LiNi_0.7_Co_0.15_Mn_0.15_O_2_ samples (Figs 3b, 4b,c and 5). The results show the spontaneously deposited CEI somewhat stabilizes during repeated cycling up to 4.5 V and to some extent suppresses the active mass dissolution and structural reconstruction at the particles surface, in good agreement with the impedance data in Fig. 3g,h. The evolution of CEI in thickness is similar to the observation of solid-electrolyte interphase growth on graphite^43^.

It is worth noting that the contribution of conductive carbon additives to the surface chemistry is not limited to composite cathode electrodes. Although different (oxidation and reduction) reactions are involved, the puzzling similar interphasial species identified on cathodes and anodes^4^,^5^,^9^,^14^,^17^,^18^,^24^,^30^,^33^ might be in part explained by the use of conductive carbon on both sides. The consistent open-circuit voltage (around 3 V) empirically observed for a broad spectrum of cathode and anode materials against Li metal before cell operation may also be related to the chemical reactivity of conductive carbon towards LiPF_6_-containing nonaqueous electrolytes, which spontaneously produces similar surface deposits on different electrode materials. In addition, the involvement of conductive carbon in the formation and evolution of cathode interphases is quite complex, and this study simply provides an intriguing glance into this phenomenon. To begin with, carbon generally possesses widely varying surface area and a diversity of surface chemical functional groups depending on the manufacturing processes. This can lead to unexpected interactions with the electrolyte and the CEIs generated may not be the same as that described here for different types of carbon additives in various cell chemistries. Further, the passivation effect of the carbon-driven CEI in simple LiPF_6_/EC-DEC solutions is far from ideal. The anodic instability of conductive carbon, including PF_5_^−^ intercalation^30^,^44^,^45^ and direct oxidation^31^ at voltages approaching 5 V vs Li/Li^+^, also becomes detrimental to cyclability. Modifications on the electrolyte can produce more robust CEIs^4^,^28^,^29^, but the detailed functioning mechanisms of these changes are not well understood. Finally, the current study monitors only the CEI evolution in terms of thickness once the electrochemical reactions initiate, rather than the chemical composition and other important properties that are also relevant to the cell performance. Thus, future studies are needed to comprehensively delineate the role of carbon in affecting the surface chemistry of composite electrodes and electrochemical properties of Li-based batteries, which should assist the development of *in situ* passivation on high-voltage cathode materials.

In summary, our findings reveal the dynamic changes of interphases involving complex, intertwined ‘communications' between the active cathode material (LiNi_0.7_Co_0.15_Mn_0.15_O_2_) and conductive carbon additives in lithium-ion batteries, and their substantial repercussion on the electrochemical properties. Notably, the majority of studies on cathode interphases thus far underestimate the influences from carbon, thereby introducing ambiguities and inconsistencies. Although some have pointed out the need to study a model electrode material surface without the carbon additives and binder^14^,^17^, in the practical sense, the interphase behaviour only present when the active material and carbon coexist needs also to be emphasized. For example, the interactions of electrolyte additives with carbon itself and/or carbon-driven interphases generated spontaneously in nonaqueous electrolytes may have a role in their effectiveness. The development of tunable and well-controlled *in situ* surface passivation on electrodes by carbon might also be an alternative approach to mitigate the surface side reactions of high-voltage, high-energy-density cathode materials in lithium-based chemistries, provided that surface functionalization of conductive carbon materials is low-cost and easy-to-use. Conversely, such efforts should at the very least serve to understand and eliminate existing unwanted molecular interactions between the electrolyte and conductive carbon (for example, graphene). Finally, we underscore the benefits of surface-sensitive techniques (such as TOF-SIMS) that enable the spatially selective observation of composite electrodes in understanding the dynamic nature of electrode interphases formation and evolution before and during electrochemical reactions.

## Methods

### Preparation of LiNi_0.7_Co_0.15_ Mn_0.15_O_2_

LiNi_0.7_Co_0.15_ Mn_0.15_O_2_ powder was prepared by a two-step method: the transition-metal co-precipitation route and subsequent annealing-induced lithiation process. In the first step, NiSO_4_·6H_2_O, CoSO_4_·7H_2_O, and MnSO_4_·H_2_O were dissolved in distilled water at a combined concentration (molar ratio of Ni:Co:Mn=70:15:15) of 1.0 mol l^−1^. Then the mixed metal solution was pumped into a 2.5 l continuously stirring tank reactor, along with optimized amounts of saturated NH_4_OH and KOH aqueous solutions fed separately into the tank under protective gas. During the reaction, the pH (11–11.5), the stirring rate (800–1,000 r.p.m.), the temperature (50–60 °C), and the residence time (4–12 h) were controlled and monitored carefully. After the co-precipitation treatment, the resulting product Ni_0.7_Co_0.15_Mn_0.15_(OH)_2_ was washed thoroughly before drying at 100 °C under vacuum overnight. The LiNi_0.7_Co_0.15_ Mn_0.15_O_2_ powder was then obtained by heating the as-prepared compound mixed with LiOH·H_2_O at a molar ratio of 1:1.03 at 800 °C for 15 h in air. The mixture was preheated at 500 °C for 5 h before the final heating step.

### Electrode fabrication and electrochemical testing

The electrochemical properties of the LiNi_0.7_Co_0.15_ Mn_0.15_O_2_ materials were studied with CR2032-type coin cells. For regular cell testing, the active material, conductive carbon additive (Super P, one type of carbon black), and binder (polyvinylidene fluoride) with the weight ratio of 80:10:10, respectively, were mixed and the resulting slurry was coated onto an aluminum foil with ∼4 mg cm^−2^ active material loading. A weight ratio of 88:1:11 was also used during the aging study to investigate the effect of carbon black. The obtained composite cathodes were assembled into coin cells with Li metal as both the counter and reference electrodes in an electrolyte solution of 1 M LiPF_6_ in a mixture of EC and diethyl carbonate (1:1, v/v). Galvanostatic discharge–charge experiments were registered on a 96-channel battery cycler (Arbin Instruments) between 3.0 and 4.5 V vs Li^+^/Li at room temperature. Impedance spectra were recorded on equilibrated cells stabilized after discharging to 3.0 V with the drift in open-circuit voltage no greater than 1 mV for 3 h, using an impedance spectrometer (Solartron 1260 A). In the impedance measurements, the a.c. frequency varied from 0.10 MHz to 2 mHz with a signal amplitude of 5 mV.

### Materials characterization

Powder XRD was carried out on a MiniFlex 600 X-ray diffractormeter (Rigaku) with Cu K_*α*_ (*λ*=1.54184 Å) radiation. Scans were conducted at 5–80° at a scan rate of 0.5° min^−1^. Chemical compositions of the LiNi_0.7_Co_0.15_ Mn_0.15_O_2_ samples were determined by an inductively coupled plasma optical emission spectrometer (Varian 715 ES). A NOVA 2000 surface area analyser (Quantachrome Instruments) was employed to obtain the Brunauer-Emmett-Teller surface area of the as-prepared samples. Images of pristine LiNi_0.7_Co_0.15_ Mn_0.15_O_2_ powder and cycled composite electrodes were taken on a Quanta 650 ESEM field-emission scanning electron microscope (FEI) and a S5500 SEM/STEM scanning transmission electron microscope (Hitachi). An aberration-corrected field-emission JEM-2100F scanning transmission electron microscope (JEOL) was also used to identify the local surface structure of cycled cathode particles at atomic resolution operated at 200 kV. STEM images were observed with a probe convergence angle of 25 mrad and the HAADF detector were used with an angle of 163 mrad. XPS measurements were performed on an Axis Ultra DLD spectrometer (Kratos) using Al Kα radiation (1486.6 eV) as the excitation source. High-resolution scanning was taken near nickel, cobalt, manganese, fluorine, oxygen, carbon and lithium regions (0.1 eV step). XPS data of NiF_2_ (Matrix Scientific 99%) was also collected during the experiment. The pressure of the analysis chamber was maintained at around 2 × 10^−9^ mbar and the binding energy was calibrated with C 1s 248.8 eV. A TOF.SIMS 5 spectrometer (ION-TOF GmbH) was used for the TOF-SIMS studies. The analysis chamber was maintained in ultra-high vacuum at a pressure below 2 × 10^−9^ mbar. All detected secondary ions of interest had a mass resolution >5,000 and possessed negative polarity. A pulsed 30 keV Bi_1_^+^ (20 ns) ion beam set in either the High Current mode or Burst Alignment mode (with seven bursts) was applied for depth profiling or high lateral resolution mapping (<200 nm) analysis, respectively. The typical analysed area was 100 × 100 μm. A 500 eV Cs^+^ ion beam (sputtering rate estimated at ∼0.03 nm s^−1^, calibrated by a pristine LiNi_0.7_Co_0.15_Mn_0.15_O_2_ composite electrode) was used for sputtering of the cycled composite electrodes or shallow milling of the cross-sectioned samples with a typical sputtered area of 300 × 300 μm. During the XPS and TOF-SIMS analyses, all samples were rinsed in dimethyl carbonate and dried in an argon-filled glovebox, and then transferred to the instruments with a novel air-free interface (U.S. Patent Application Serial No. 14/445,650 filed 29 July 2014).

### Data availability

The data that support the findings of this study are available from the corresponding author upon request.

## Additional information

**How to cite this article:** Li, W. *et al*. Dynamic behaviour of interphases and its implication on high-energy-density cathode materials in lithium-ion batteries. *Nat. Commun.* **8**, 14589 doi: 10.1038/ncomms14589 (2017).

**Publisher's note:** Springer Nature remains neutral with regard to jurisdictional claims in published maps and institutional affiliations.

## Supplementary Material

Supplementary Information Supplementary Figures 1-30 and Supplementary Tables 1-8
