# Supplementary material for: Dynamic behaviour of interphases and its implication on high-energy-density cathode materials in lithium-ion batteries
Source: Nat Commun. 2017 Apr 26;8:14589. doi: 10.1038/ncomms14589 (PMC5414066; doi:10.1038/ncomms14589)
Supplement: Supplementary Information — Supplementary Figures 1-30 and Supplementary Tables 1-8 [file ncomms14589-s1.pdf]

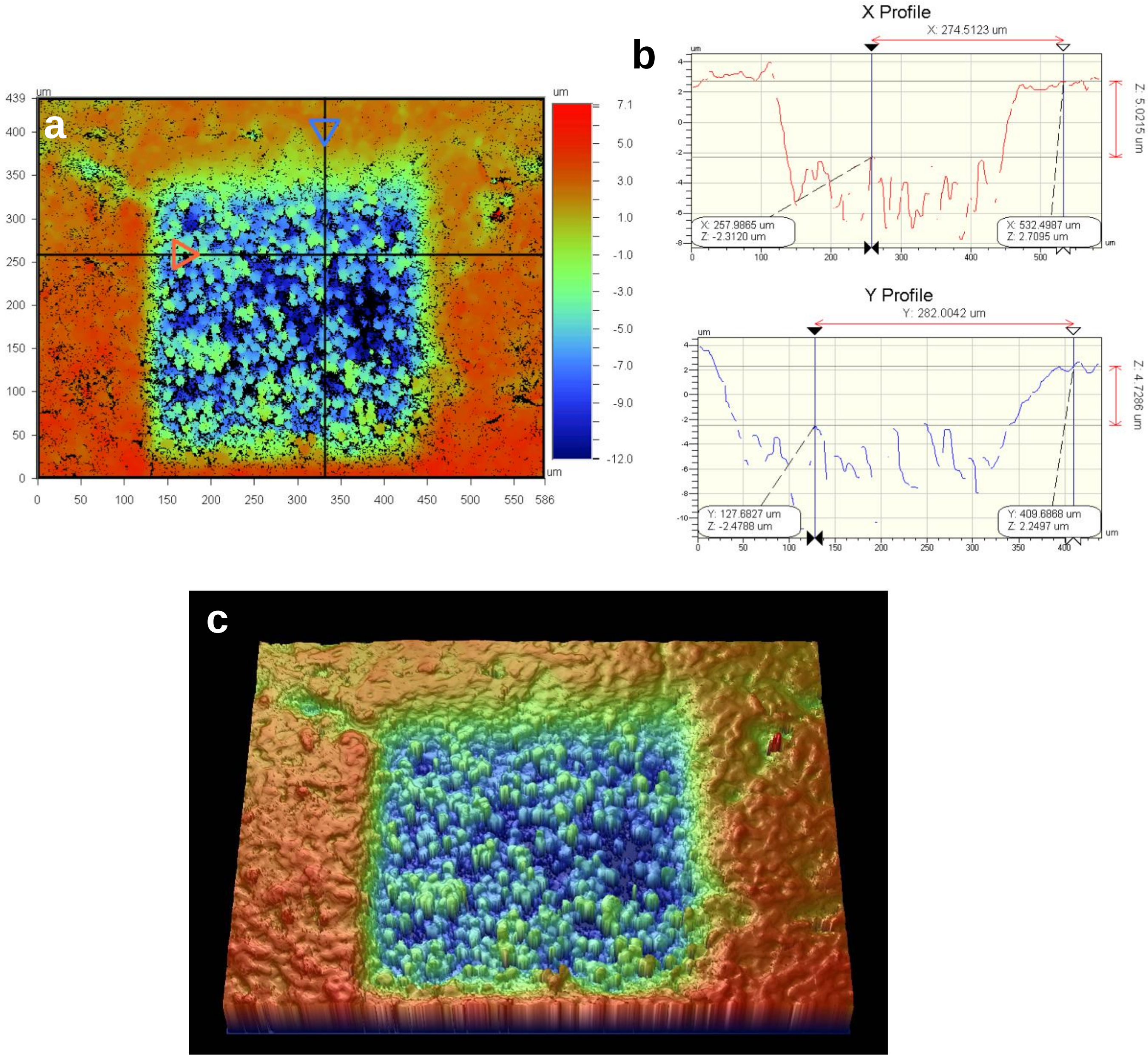

Supplementary Figure 1: Calibration of the Cs^+ sputtering rate on composite $LiNi_{0.7}Mn_{0.15}Co_{0.15}O_2$ electrodes (500 eV ion energy, ~40 nA measured sample current): (a) Optical profilometry mapping of a Cs^+ sputtered area (300 × 300 μm^2) for 44 hours on the 18-20 μm pristine electrode. (b) Vertical profiles of the optical map along the planes indicated in (a). The massive surface corrugation increase within the sputtered region with respect to the adjacent area indicates a significant (~ 2 - 2.5 times) higher sputtering rate of the carbon additive/binder with respect to that of the active material. The sputtering rate of the active material is estimated at 0.03 nm s^{-1} and that of the carbon additive/binder at 0.07 nm s^{-1}. (c) 3D view of the optical map in (a).

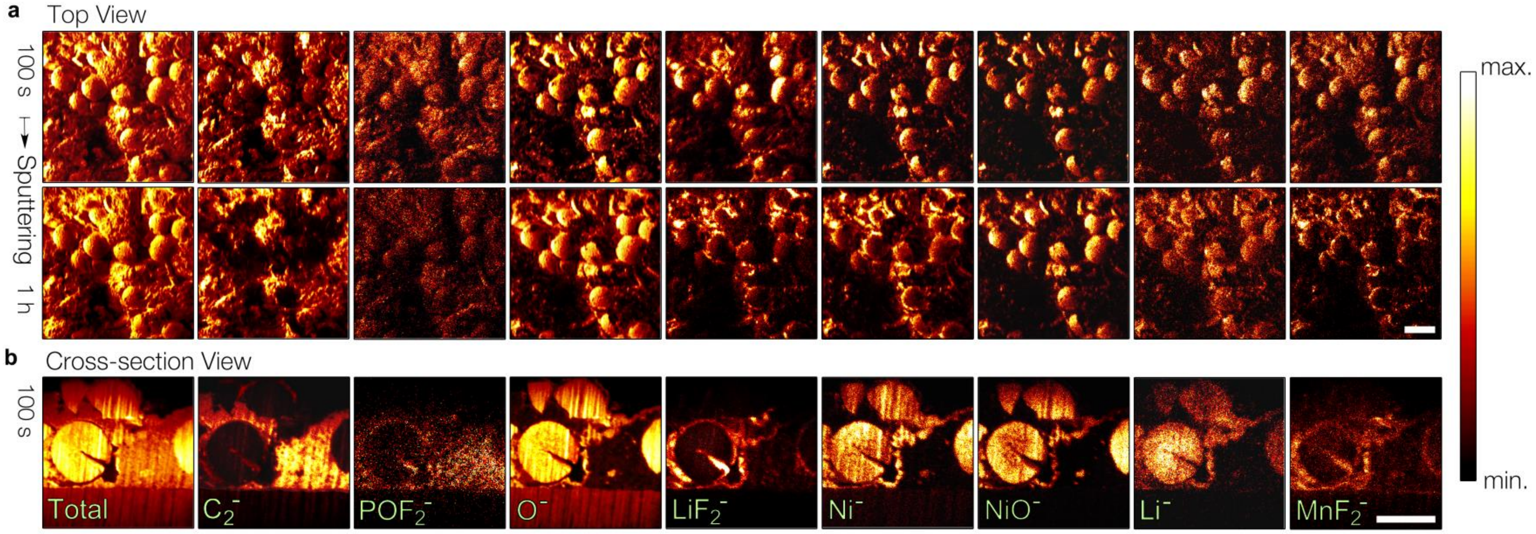

Supplementary Figure 2: Comparative high-resolution TOF-SIMS mappings (burst alignment mode) of cycled $LiNi_{0.7}Mn_{0.15}Co_{0.15}O_2$ electrode after 100 cycles at room temperature: (a) top views collected after 100 s and 1 h Cs^+ etching, while (b) is taken from the cross-sectional perspective. From the left to right, the secondary ions of interest are, respectively, 'total', C_2^-, POF_2^-, O^-, LiF_2^-, Ni^-, NiO^-, Li^-, and MnF_2. Notably, the rock-salt phase (represented by NiO^-) appears richer at the exterior of a secondary particle whereas the pristine layered (Li^-) is more concentrated at the core. The scale bars are 20 μm in both (a) and (b).

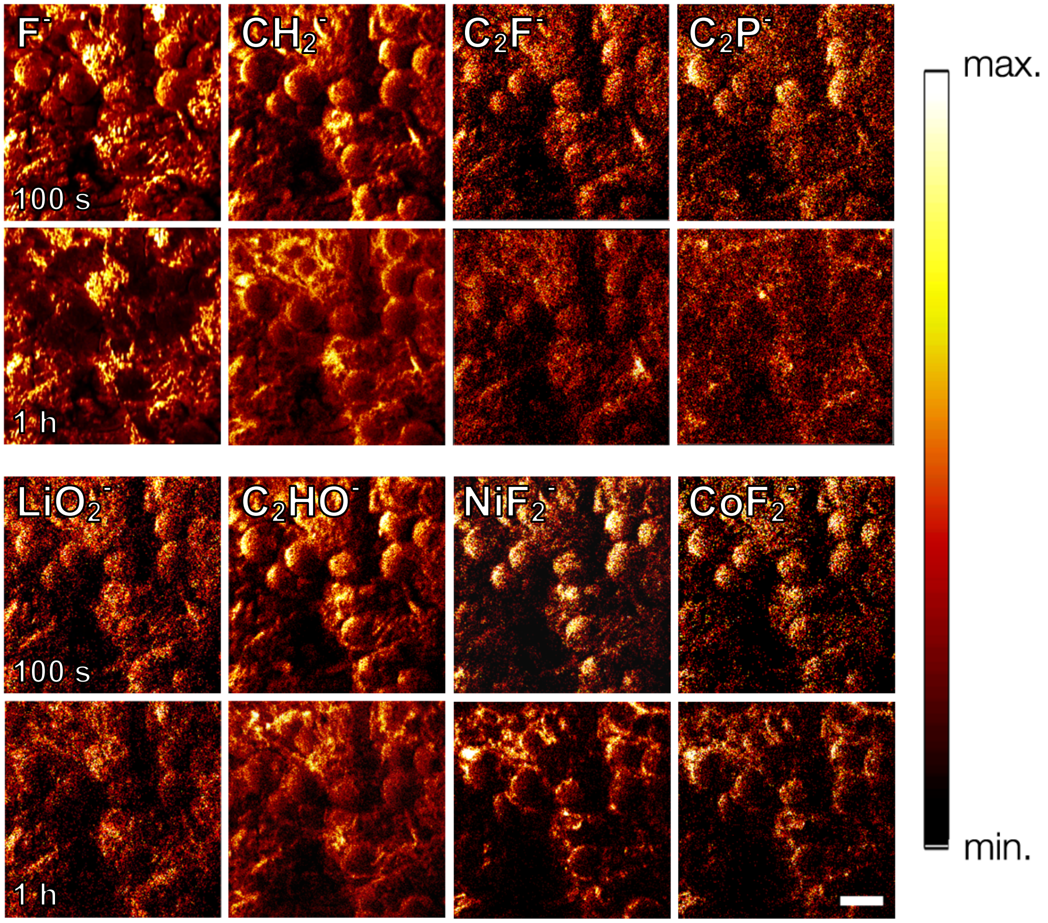

Supplementary Figure 3: Additional TOF-SIMS mapping after 100 s and 1 hour of Cs^+ sputtering, on the $LiNi_{0.7}Mn_{0.15}Co_{0.15}O_2$ composite electrodes after 100 cycles, of several species of interest, showing the interphasial species (F^-, CH_2^-, LiO_2^-, C_2F^-, C_2P^-, C_2HO^-, NiF_2^-, and CoF_2^-) evolution as function of depth. Scale bar is 20 μm.

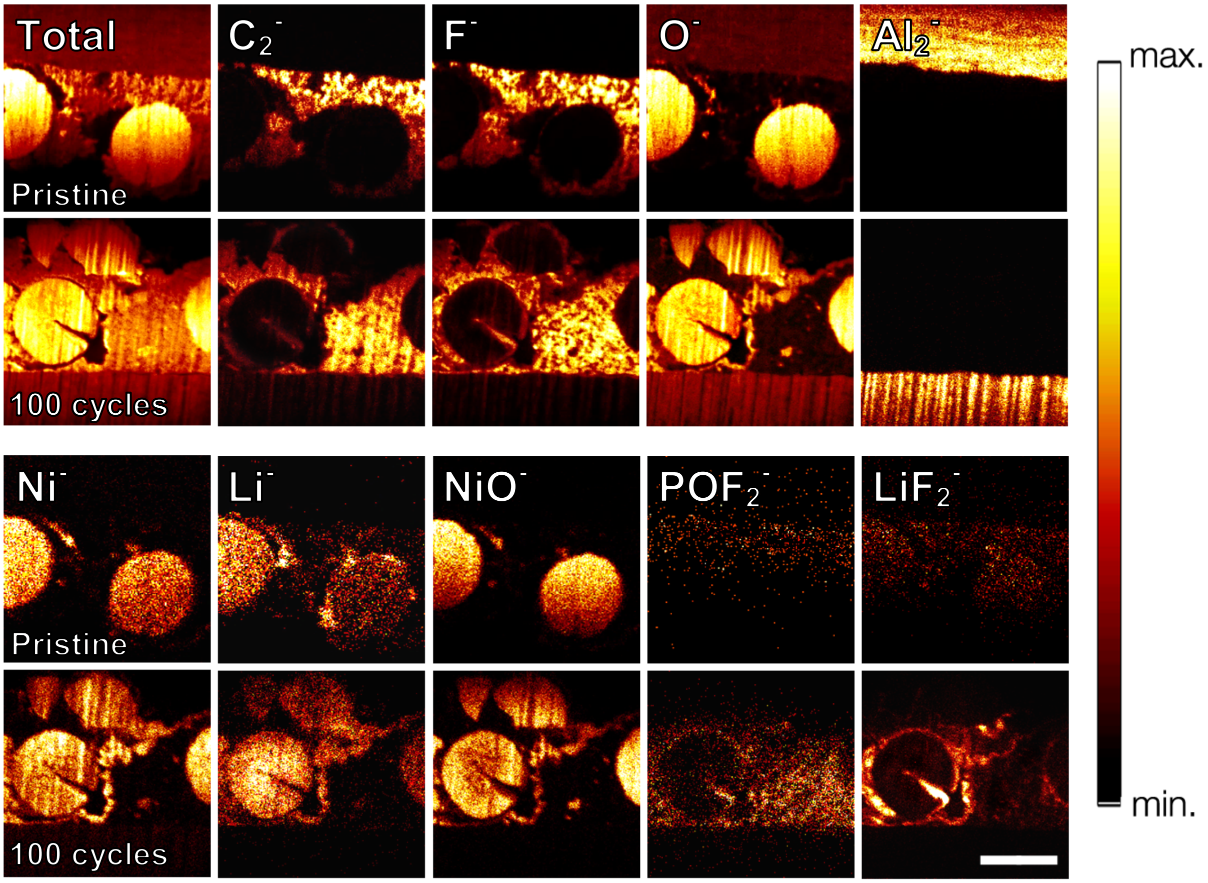

Supplementary Figure 4: Comparative cross-sectional high resolution TOF-SIMS mapping on the $LiNi_{0.7}Mn_{0.15}Co_{0.15}O_2$ pristine *vs.* 100 cycles composite electrode after 100 seconds of Cs^+ sputtering demonstrating the carbon additive/binder (C_2^-, F^-), aluminum current collector (Al_2^-), active material particles (Li^-, O^-, Ni^-, NiO^-) and interphasial species (LiF_2^-, POF_2^-) formation after cycling. Note the change in spatial distribution of Li^- and NiO^- indicative of rock-salt phase formation after cycling; also Li^-, O^-, Ni^-, and NiO^- signals are present on the carbon additive/binder surface for the cycled electrode, indicative of the active mass dissolution. Scale bar is 20 μm.

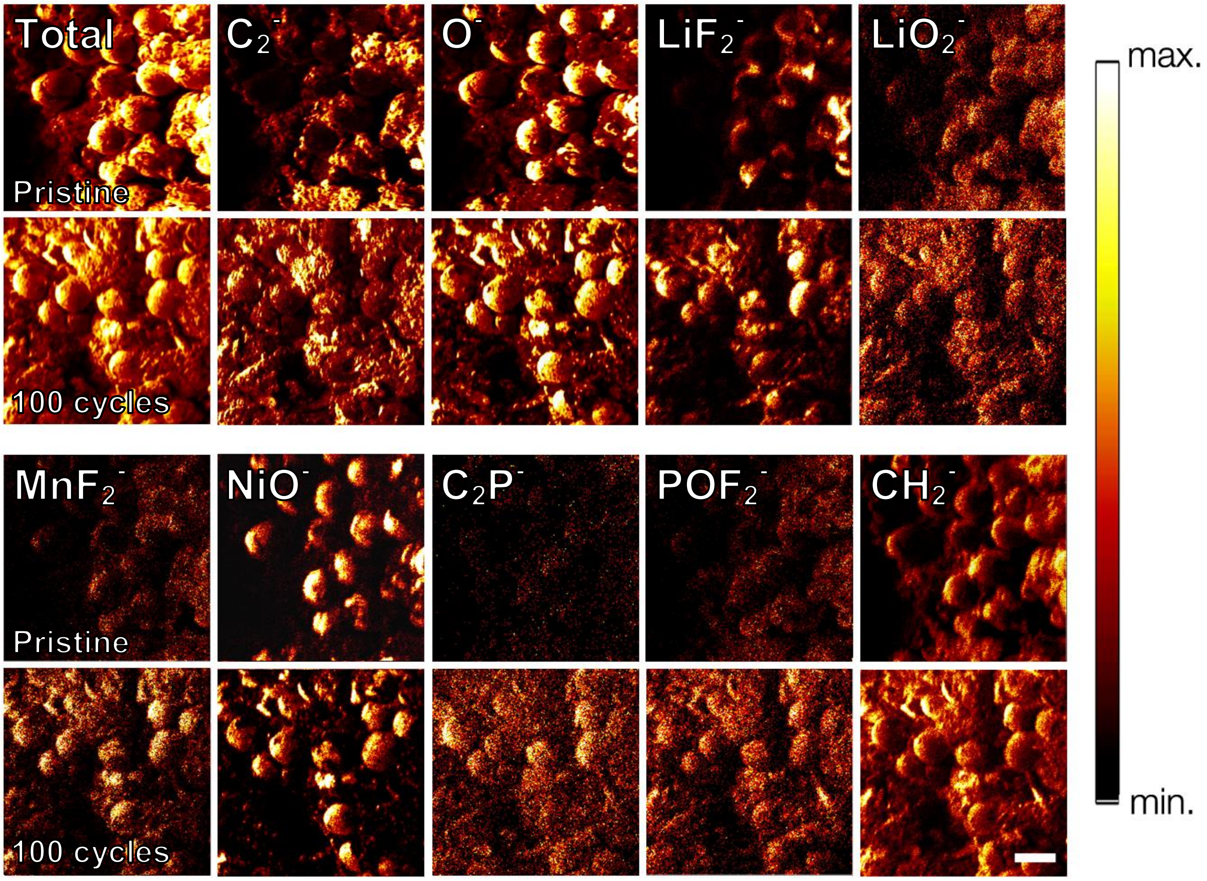

Supplementary Figure 5: Comparative high resolution TOF-SIMS mapping on the $LiNi_{0.7}Mn_{0.15}Co_{0.15}O_2$ pristine *vs.* 100 cycles composite electrode after 100 seconds of Cs^+ sputtering showing the formation of interphasial species (CH_2^-, C_2^-, LiF_2^-, C_2P^-, POF_2^-, and MnF_2^-) after cycling. Scale bar is 20 μm.

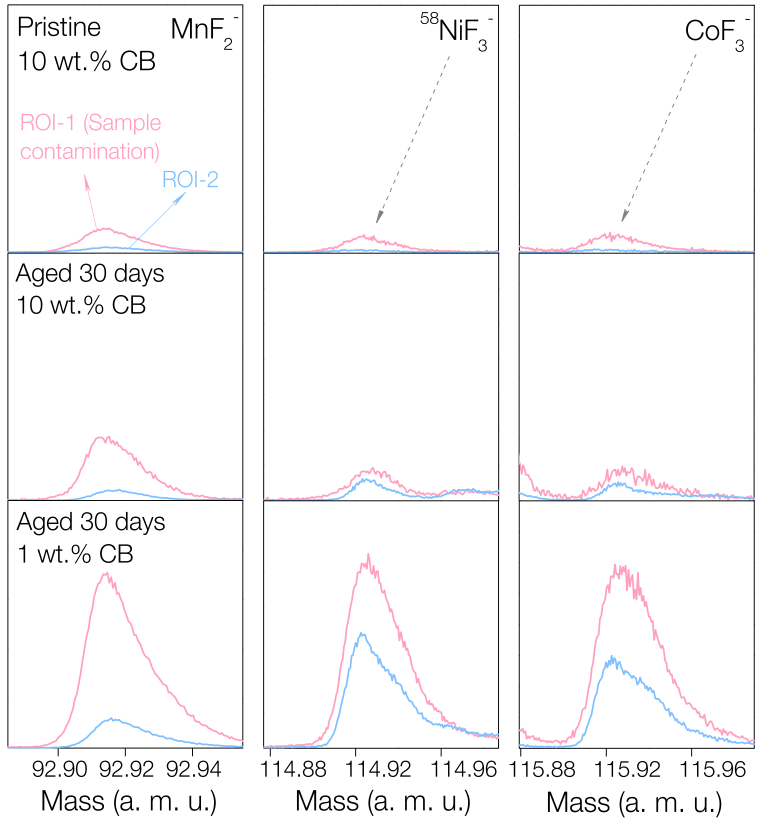

Supplementary Figure 6: TOF-SIMS spectra on pristine (10 wt.% carbon black, shortened for 'CB' in the figure), 30-day aged (1 and 10 wt.% carbon black) $LiNi_{0.7}Mn_{0.15}Co_{0.15}O_2$ electrodes, with ROI-1 and ROI-2 selection, respectively. Integration sputtering time is 600 s, with 2 scans per 10 s. The fragments of interests present are, respectively, MnF_2^-, $^{58}NiF_3^-$, and CoF_3^- from left to right. These species, along with $^{7}LiF_2^-$ and MnF_3^- in the main article, collectively represent the active mass dissolution products aggravated by acid leaching from the electrolyte. All spectra are normalized by ROI coverage and drawn to the same scale in each panel. The migration of dissolution products from the active material towards the carbon/binder is clearly shown.

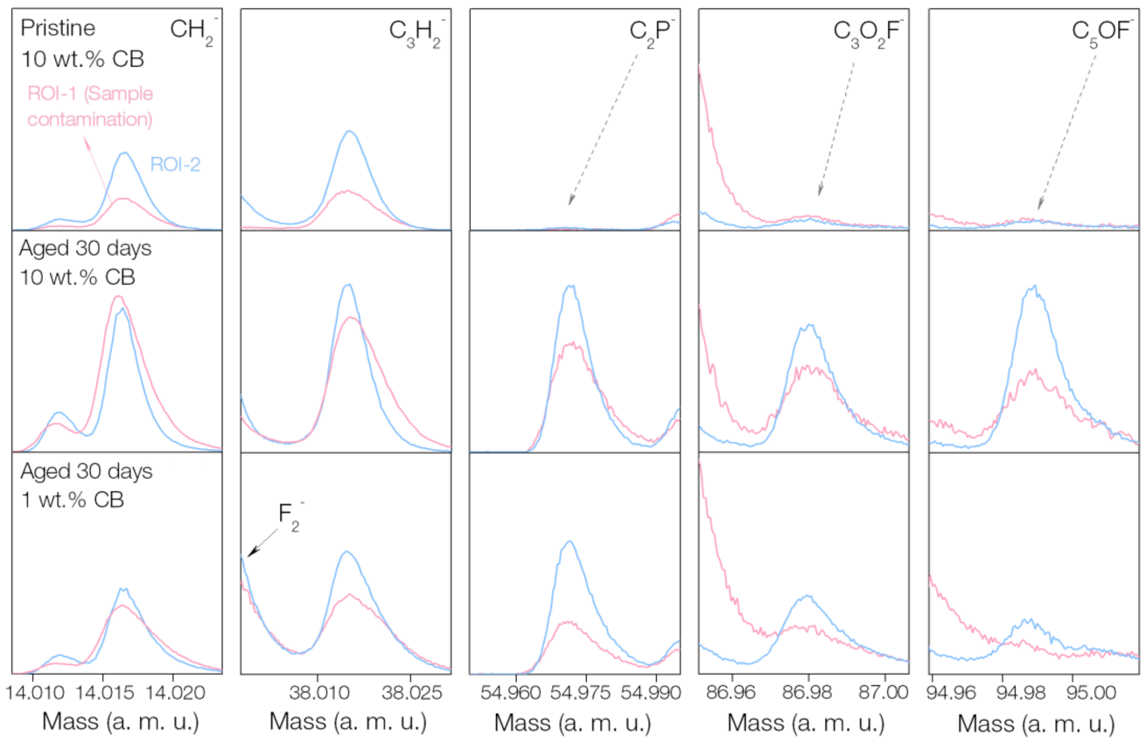

Supplementary Figure 7: TOF-SIMS spectra on pristine (10 wt.% carbon black, shortened for 'CB' in the figure), 30-day aged (1 and 10 wt.% carbon black) $LiNi_{0.7}Mn_{0.15}Co_{0.15}O_2$ electrodes, with ROI-1 and ROI-2 selection, respectively. Integration sputtering time is 600 s, with 2 scans per 10 s. The fragments of interests present are, respectively, CH_2^-, $C_3H_2^-$, C_2P^-, $C_3O_2F^-$, and C_5OF^- from left to right. These species, along with C_2F^- and C_3OF^- in the main article, collectively represent the spontaneously formed CEI due to the reactivity between carbon black and the electrolyte. All spectra are normalized by ROI coverage and drawn to the same scale in each panel. Note that a fair amount of CH_2^- and $C_3H_2^-$ are detected on the pristine electrode due to inevitable sample contamination. The migration of CEI species from the carbon/binder towards the active material is clearly shown.

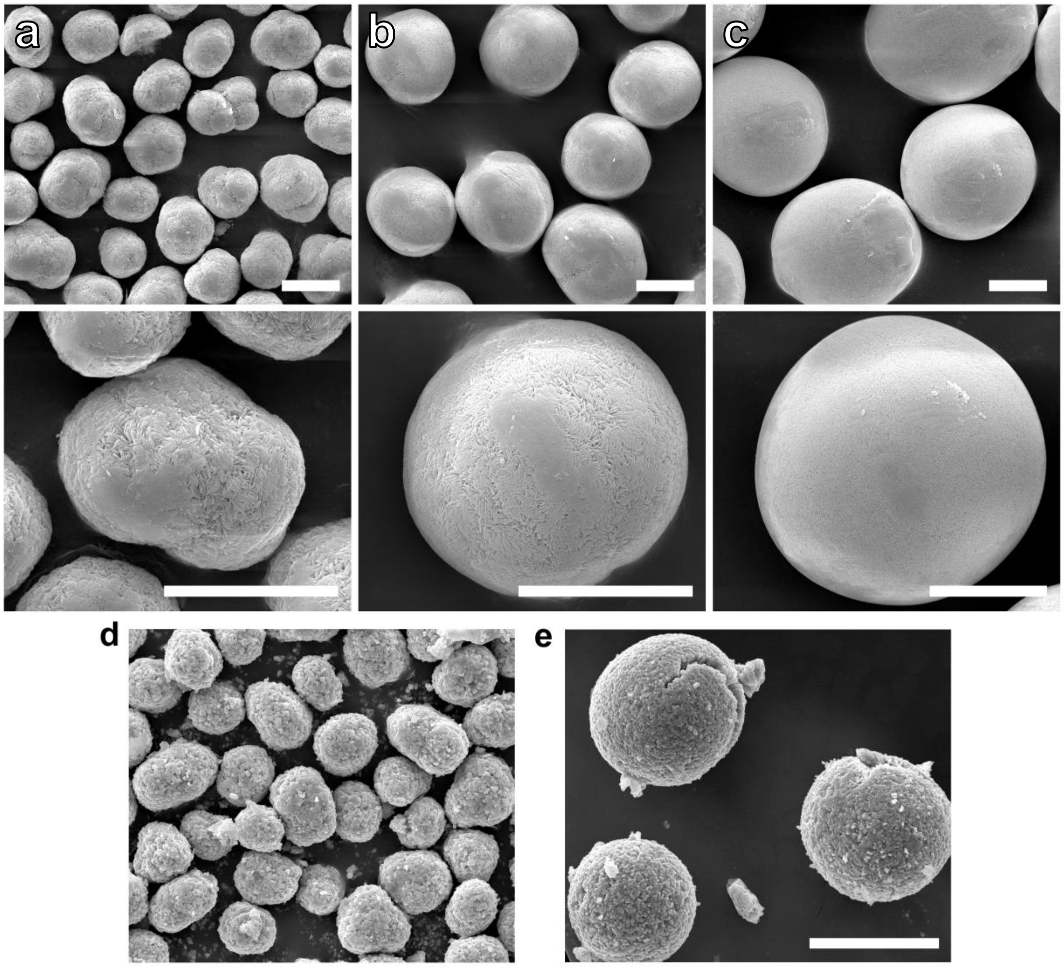

Supplementary Figure 8: SEM images of the as-prepared samples used in the current study: transition-metal hydroxide precursors, $Ni_{0.7}Co_{0.15}Mn_{0.15}(OH)_2$, with an overall spherical shape and narrow particle-size distributions of 8-10 μm (a), 12-14 μm (b) and 18-20 μm (c), and $LiNi_{0.7}Co_{0.15}Mn_{0.15}O_2$ of 8-10 μm (d) and 18-20 μm (e). The scale bars are 20 μm in (a)-(c), and 10 μm in (d) and (e).

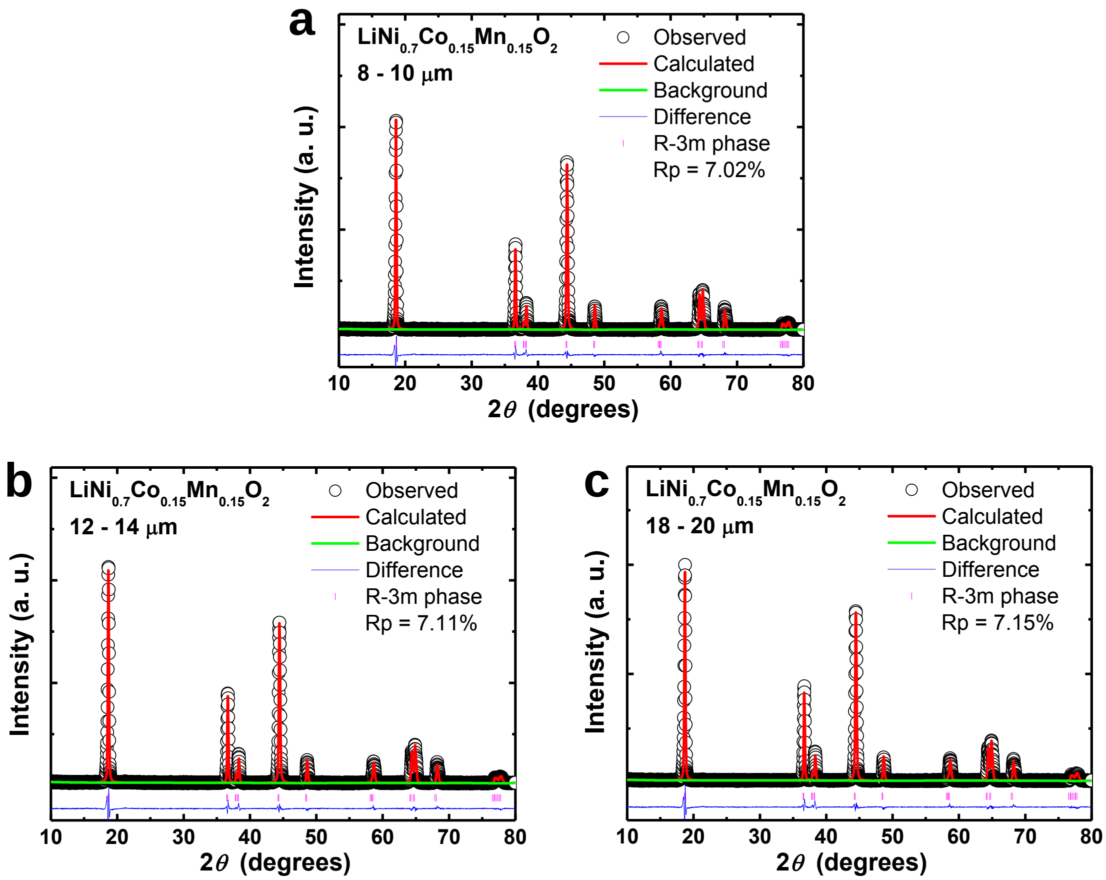

Supplementary Figure 9: Powder XRD patterns (black dots) on pristine $LiNi_{0.7}Co_{0.15}Mn_{0.15}O_2$ samples with Rietveld refinement (red lines) of different secondary particle size: (a) 8-10 μm, (b) 12-14 μm, and (c) 18-20 μm. Bragg positions are indicated by purple vertical tick marks. The difference between the calculated patterns and observed data is shown as the blue line.

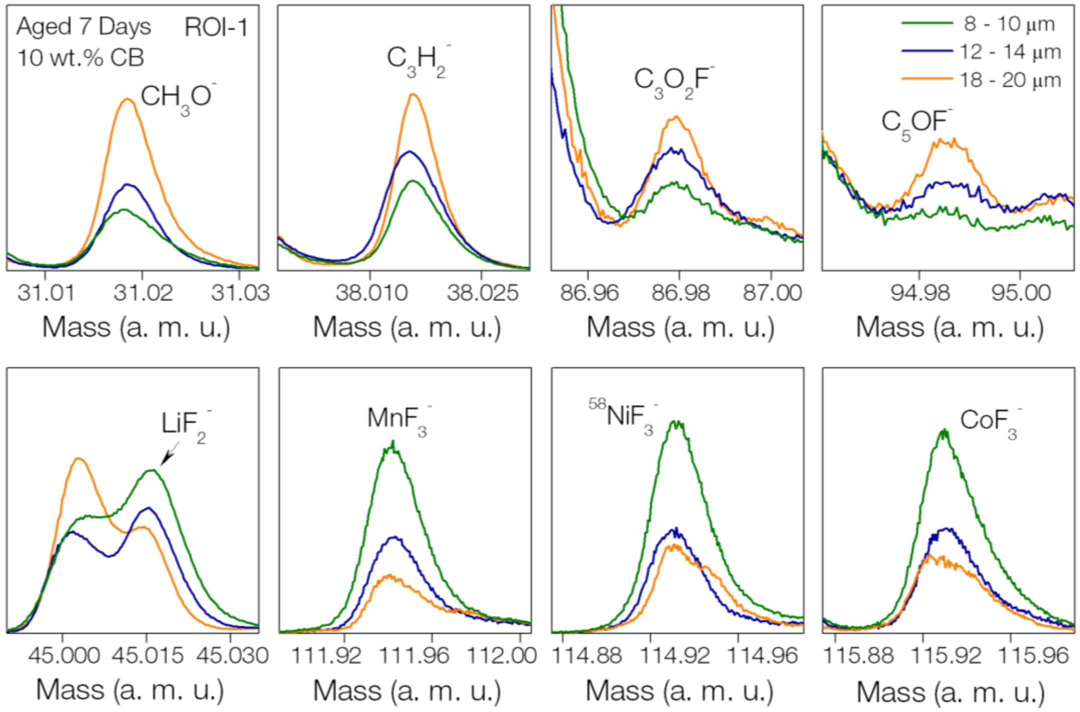

Supplementary Figure 10: TOF-SIMS spectra of several fragments representing the CEI and dissolution products (ROI-1 applied and normalized by ROI coverage; integrated over 1,000 s of Cs^+ sputtering, 10 s interval, 2 scans at each step) on aged $LiNi_{0.7}Mn_{0.15}Co_{0.15}O_2$ particles after 7 days. The protection of CEI on the active material against acid leaching is readily noticeable among the three samples.

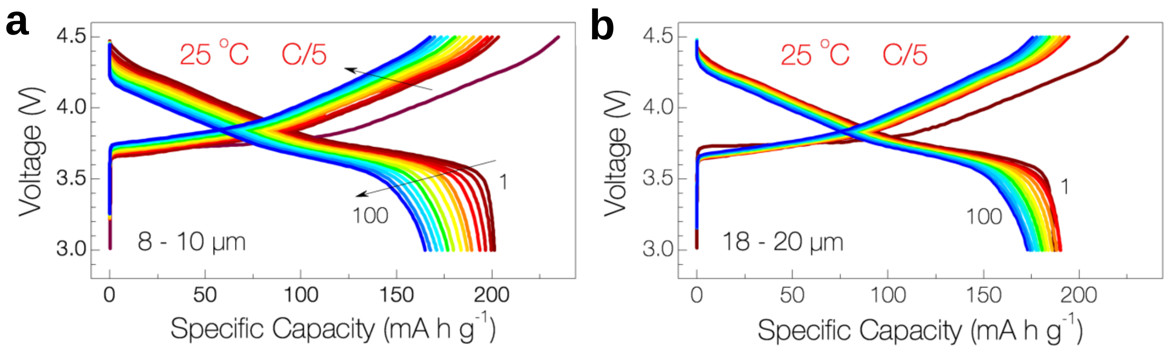

Supplementary Figure 11: Galvanostatic charge-discharge tests of $LiNi_{0.7}Mn_{0.15}Co_{0.15}O_2$ electrodes at room temperature: (**a**) 8–10 μm and (**b**) 18–20 μm during 100 cycles at C/5.

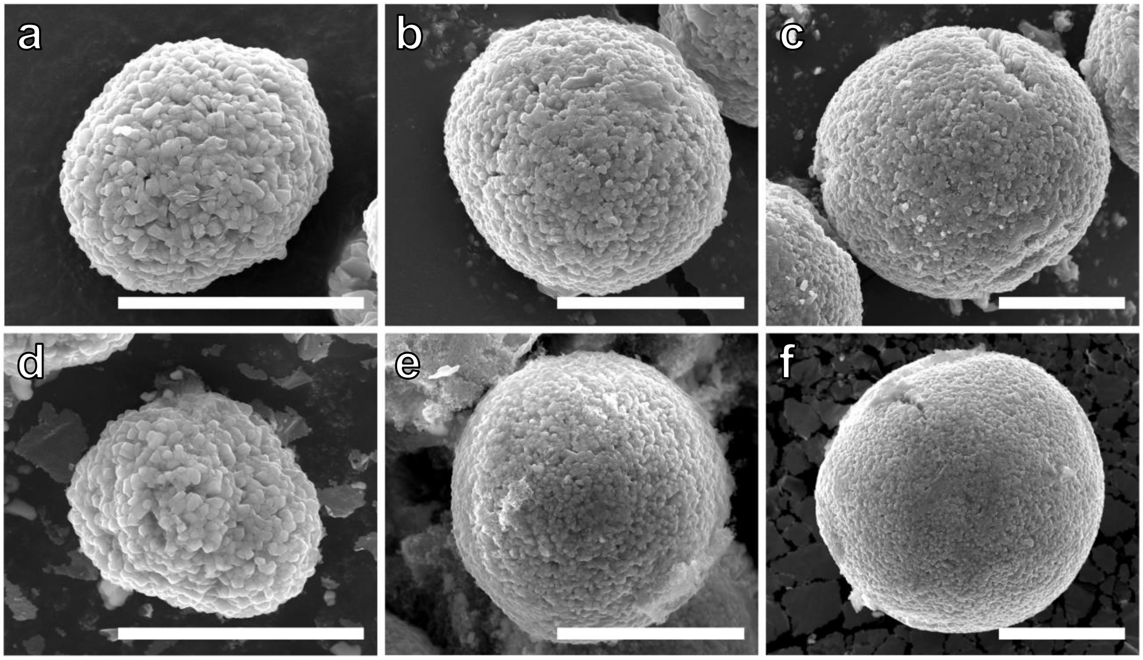

Supplementary Figure 12: SEM images of the pristine $LiNi_{0.7}Mn_{0.15}Co_{0.15}O_2$ powder with different secondary particle size: (a) 8–10 μm, (b) 12–14 μm, and (c) 18–20 μm. After 100 cycles at room temperature the particles maintain shape integrity: (d) 8–10 μm, (e) 12–14 μm, and (f) 18–20 μm. All scale bars are 10 μm.

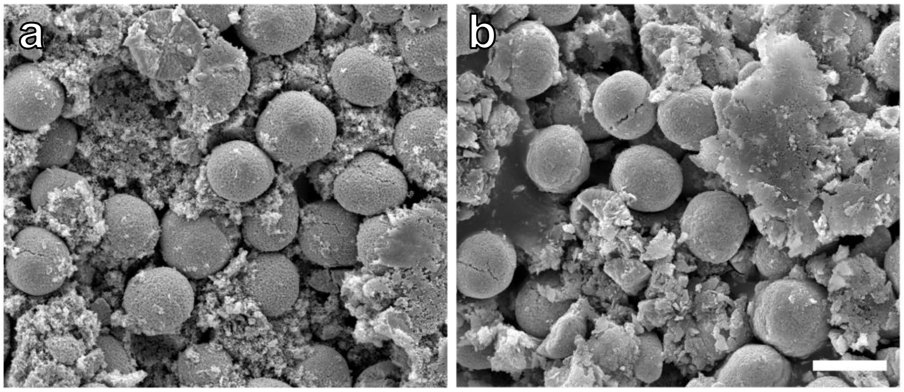

Supplementary Figure 13: SEM imaging on the 18–20 μm $LiNi_{0.7}Mn_{0.15}Co_{0.15}O_2$ composite electrodes in (a) pristine state and (b) after 100 cycles. The structural integrity is largely preserved at this cycling stage. Some particle fracture/dissolution is observed in the cycled electrode. Scale bar is 20 μm.

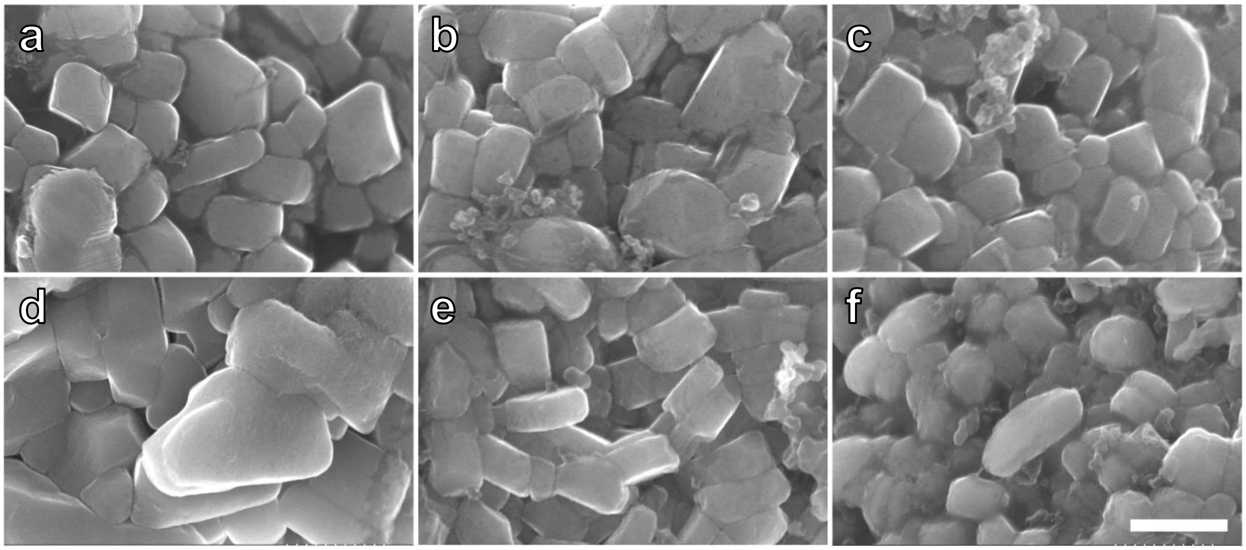

Supplementary Figure 14: SEM imaging of the primary particles in the (a) 8–10 μm, (b) 12–14 μm, and (c) 18–20 μm $LiNi_{0.7}Mn_{0.15}Co_{0.15}O_2$ pristine composite electrodes. The primary particle size appears similar with different secondary particle size. After 100 cycles the interphase formation is not clearly visible: (d) 8–10 μm, (e) 12–14 μm, and (f) 18–20 μm. Scale bar is 500 nm.

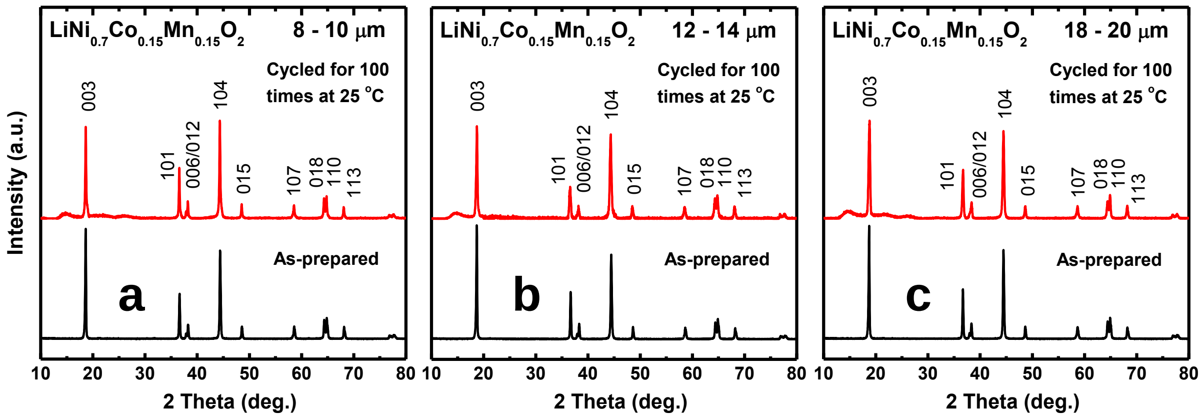

Supplementary Figure 15: A comparison of XRD data between the pristine and cycled $LiNi_{0.7}Co_{0.15}Mn_{0.15}O_2$ (100 cycles at room temperature) samples: (a) 8-10 μm, (b) 12-14 μm, and (c) 18-20 μm. As a rule, ratio of the (003) *vs.* (104) peaks is a good indicator of the rock-salt phase (NiO) generation; a larger decrease in the value is associated with more NiO phase formation.

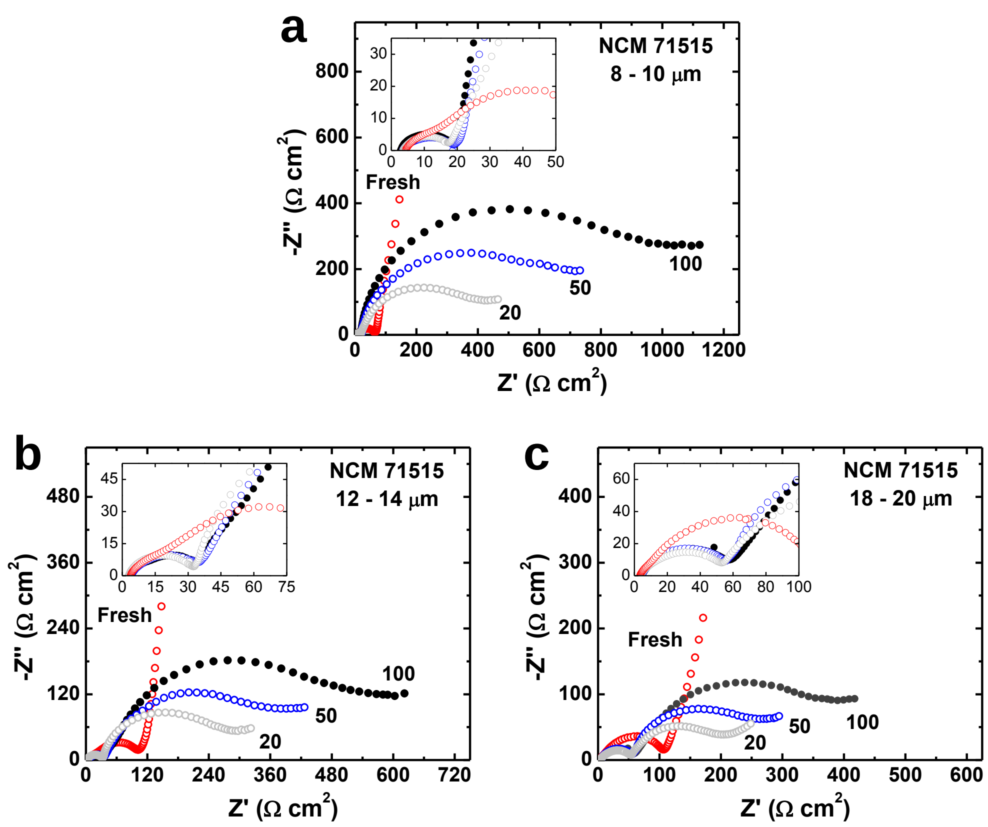

Supplementary Figure 16: Nyquist plots showing the impedance evolution of the $LiNi_{0.7}Co_{0.15}Mn_{0.15}O_2$ electrodes as a function of cycling numbers: (a) 8 – 10 μm, (b) 12 14 μm, (c) 18 – 20 μm. Inset demonstrates the magnified region of the high-frequency semicircles in the main figure.

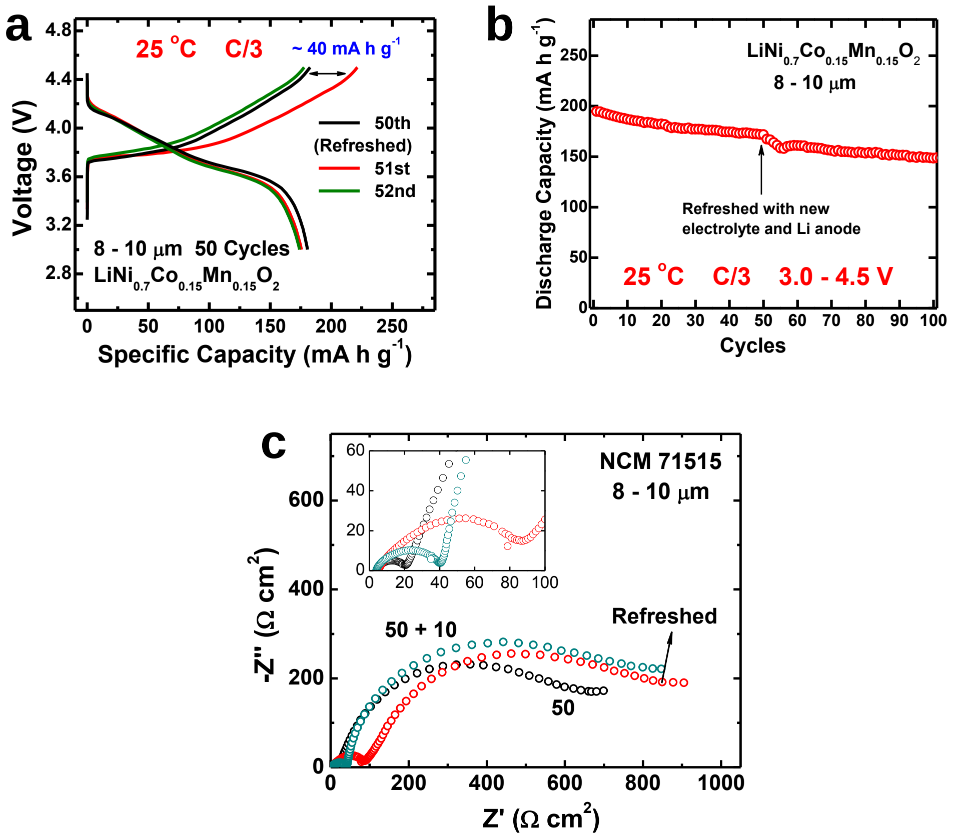

Supplementary Figure 17: Evolution of cell performance and impedance before and after Li anode and electrolyte refreshing: (a) galvanostatic charge-discharge curves of the 8 – 10 μm $LiNi_{0.7}Co_{0.15}Mn_{0.15}O_2$ electrodes prior to and after refreshing with new electrolyte and Li anode. Large irreversible charge capacity appeared at the initial cycle after the refreshing. (b) Capacity fade as a function of cycling. In (a) and (b), no improvement in the voltage and capacity drop emerges after refreshing. (c) Evolution of the impedance data for the cell prior to and after refreshing. As seen, the surface film resistance increases dramatically (still relatively small) while the charge-transfer resistance remains virtually unchanged, which suggests the surface film on the Li anode does not significantly contribute to the sluggish Li-ion insertion/extraction kinetics (at the current cycling stage).

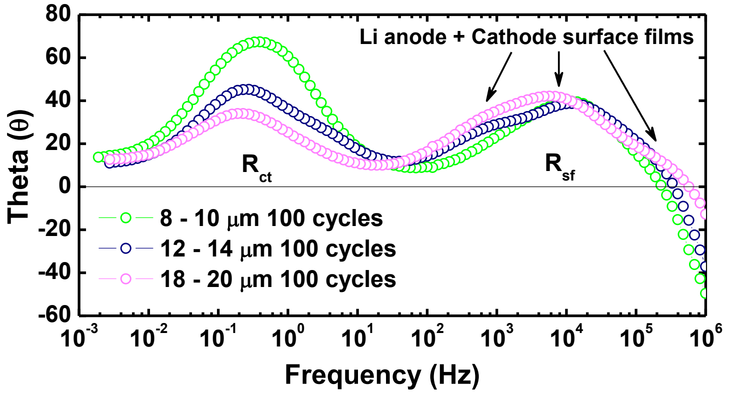

Supplementary Figure 18: Bode plot depicting the impedance evolution of the $LiNi_{0.7}Co_{0.15}Mn_{0.15}O_2$ electrodes after 100 cycles as a function of frequency. It can be seen that the first semicircle in the above Nyquist diagrams consists of multiple components, which are attributed to lithium-ion migration through the surface films formed both on the Li anode and cathode electrode surface.

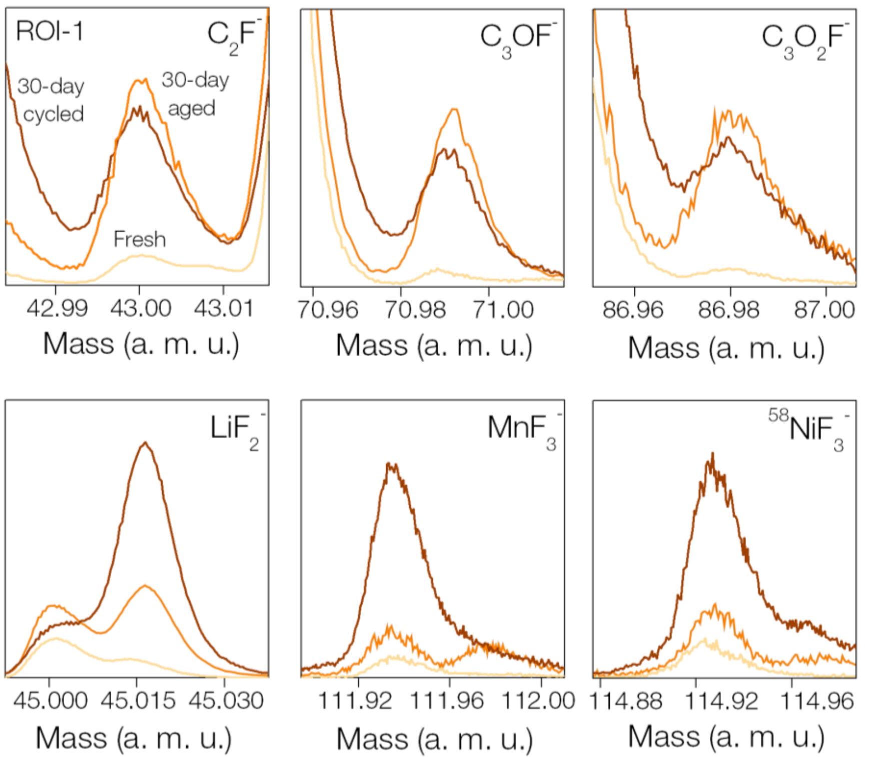

Supplementary Figure 19: TOF-SIMS spectra of several fragments representing the CEI (top row) and dissolution products (bottom row) on $LiNi_{0.7}Mn_{0.15}Co_{0.15}O_2$ electrodes after 30 days of aging or cycling at room temperature (4.5 V upper cut-off). Data collected on the pristine electrode are also shown in the figure. ROI-1 selection is applied and all spectra are normalized by ROI coverage, which are integrated over 600 s of Cs^+ sputtering with 10 s interval and 2 scans per step. It can be seen that the CEI species in part decompose during the high-voltage battery operation, while the active mass dissolution is severely aggravated.

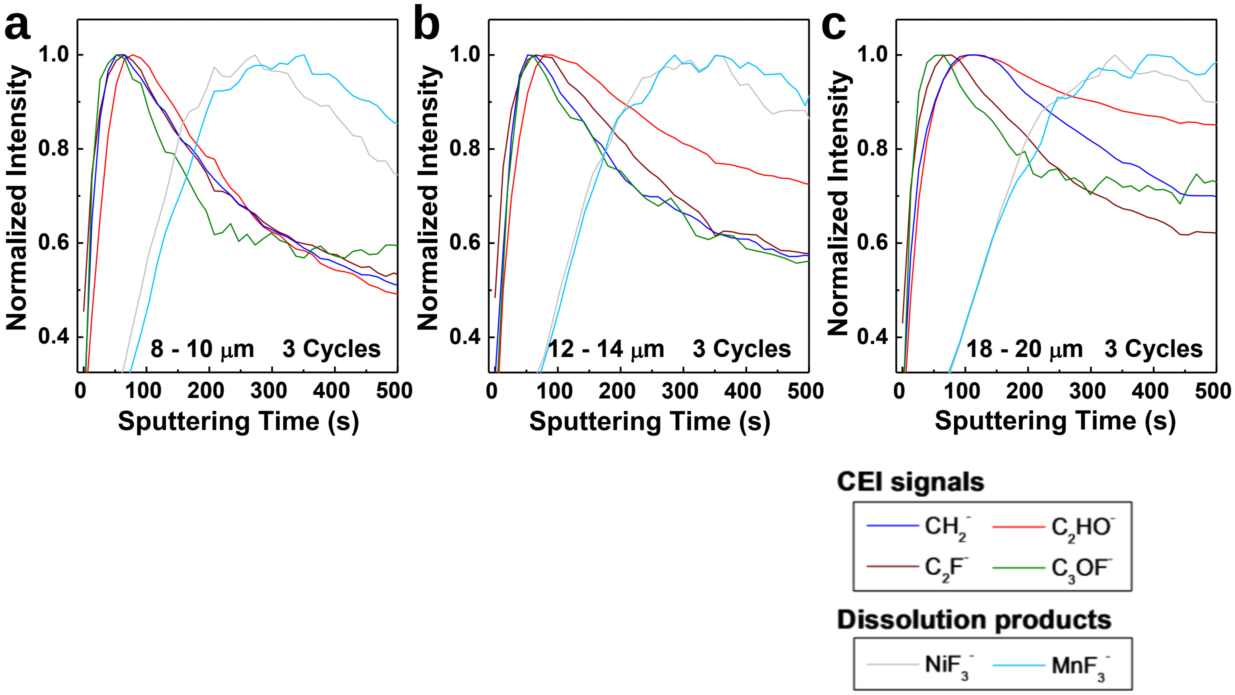

Supplementary Figure 20: Normalized (to maximum) TOF-SIMS depth profiles of short scans (500 s of Cs^+ sputtering) for the 8-10 μm (a), 12-14 μm (b), and 18-20 μm (c) composite electrodes after 3 cycles (data were collected separately at different locations on the composite electrodes and one is shown here; the same applies to Supplementary Fig. 21 and 22). The CEI formation depth, upon cell operation, is still larger with the increasing secondary particle size.

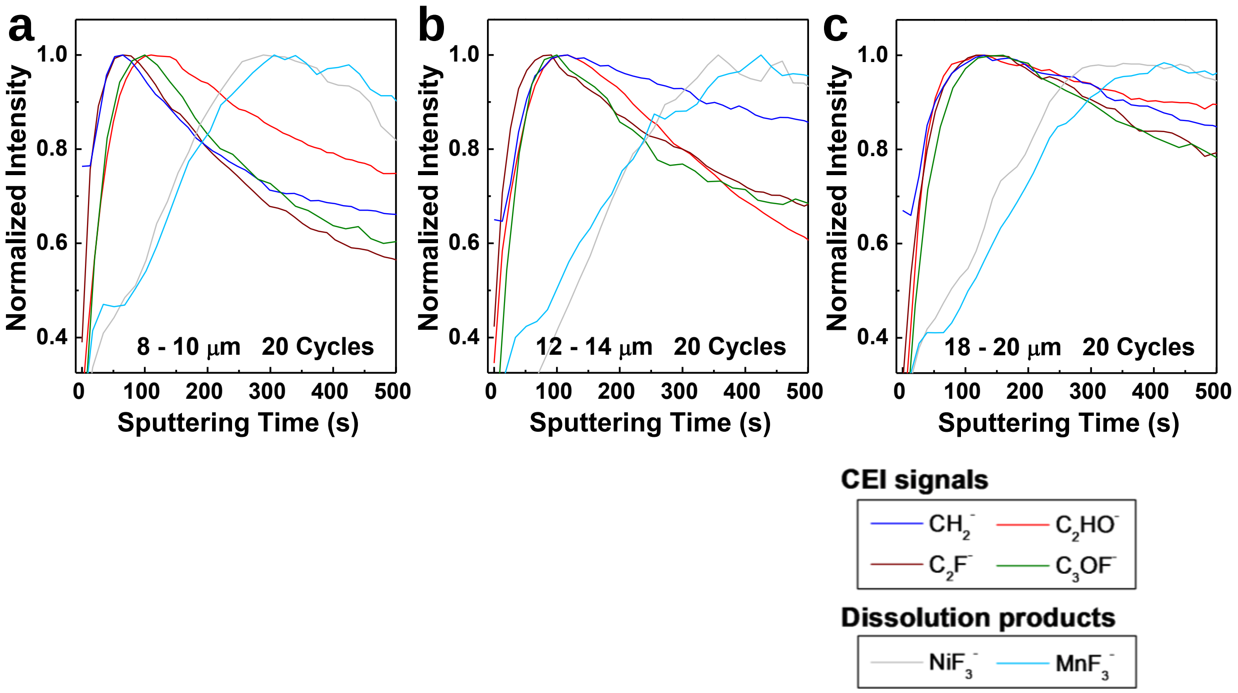

Supplementary Figure 21: : Normalized (to maximum) TOF-SIMS depth profiles of short scans (500 s of Cs^+ sputtering) for the 8-10 μm (a), 12-14 μm (b), and 18-20 μm (c) composite electrodes after 20 cycles. The CEI formation depth is larger with the increasing secondary particle size at this cycling stage.

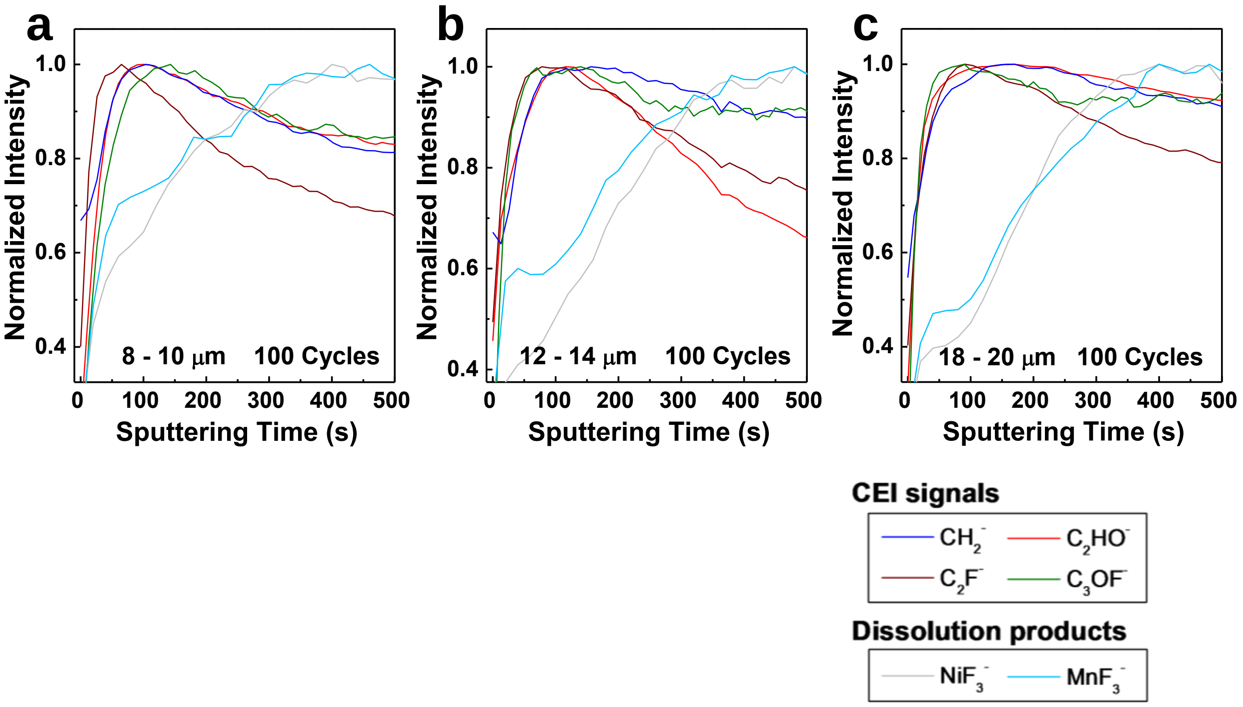

Supplementary Figure 22: Normalized (to maximum) TOF-SIMS depth profiles of short scans (500 s of Cs^+ sputtering) for the 8-10 μm (a), 12-14 μm (b), and 18-20 μm (c) composite electrodes after 100 cycles. The CEI formation depth only shows small buildup for a given electrode until this cycling stage, and is still larger with increasing secondary particle size.

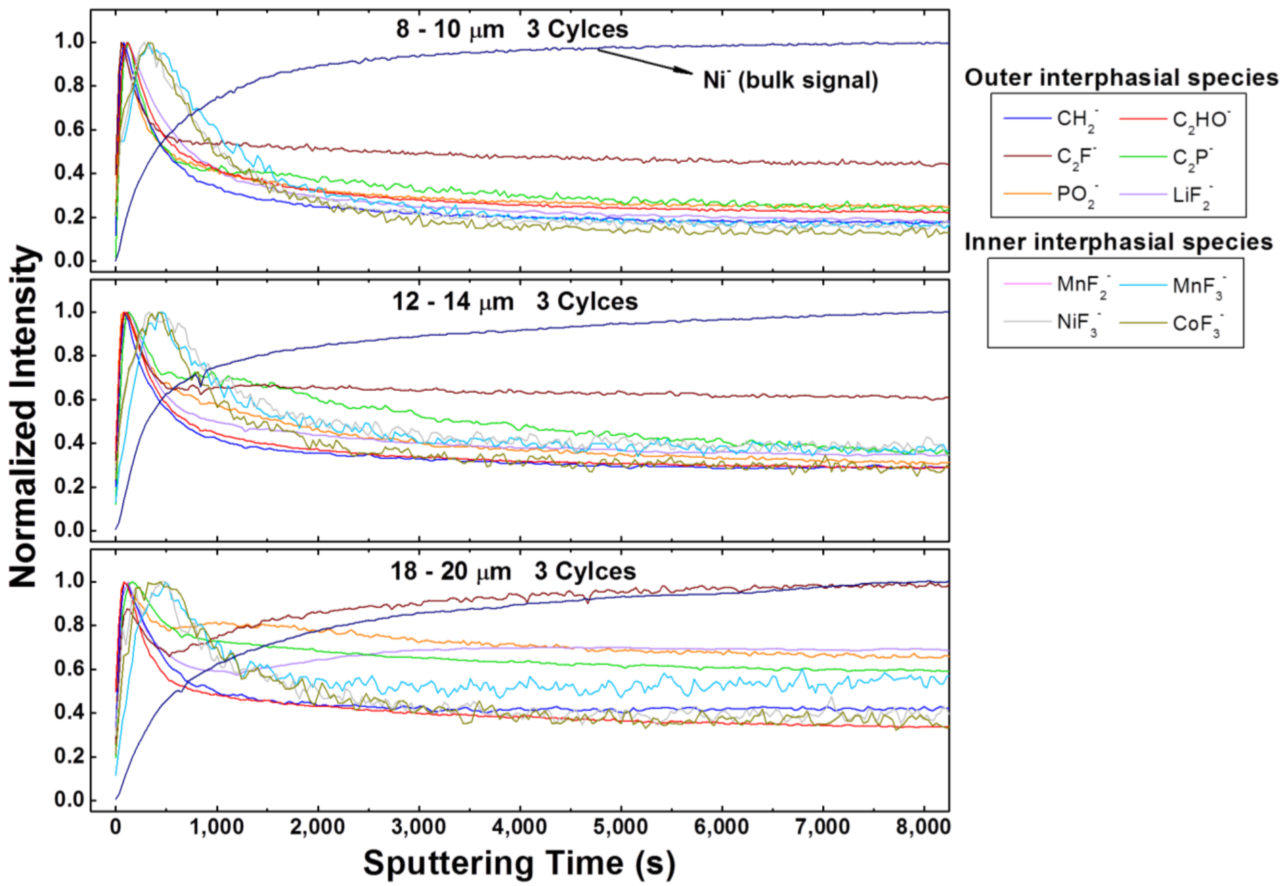

Supplementary Figure 23: Normalized (to maximum) TOF-SIMS depth profiles of outer and inner interphasial species representative secondary ion fragments for the 8-10 μm, 12-14 μm and 18-20 μm composite electrodes after 3 cycles.

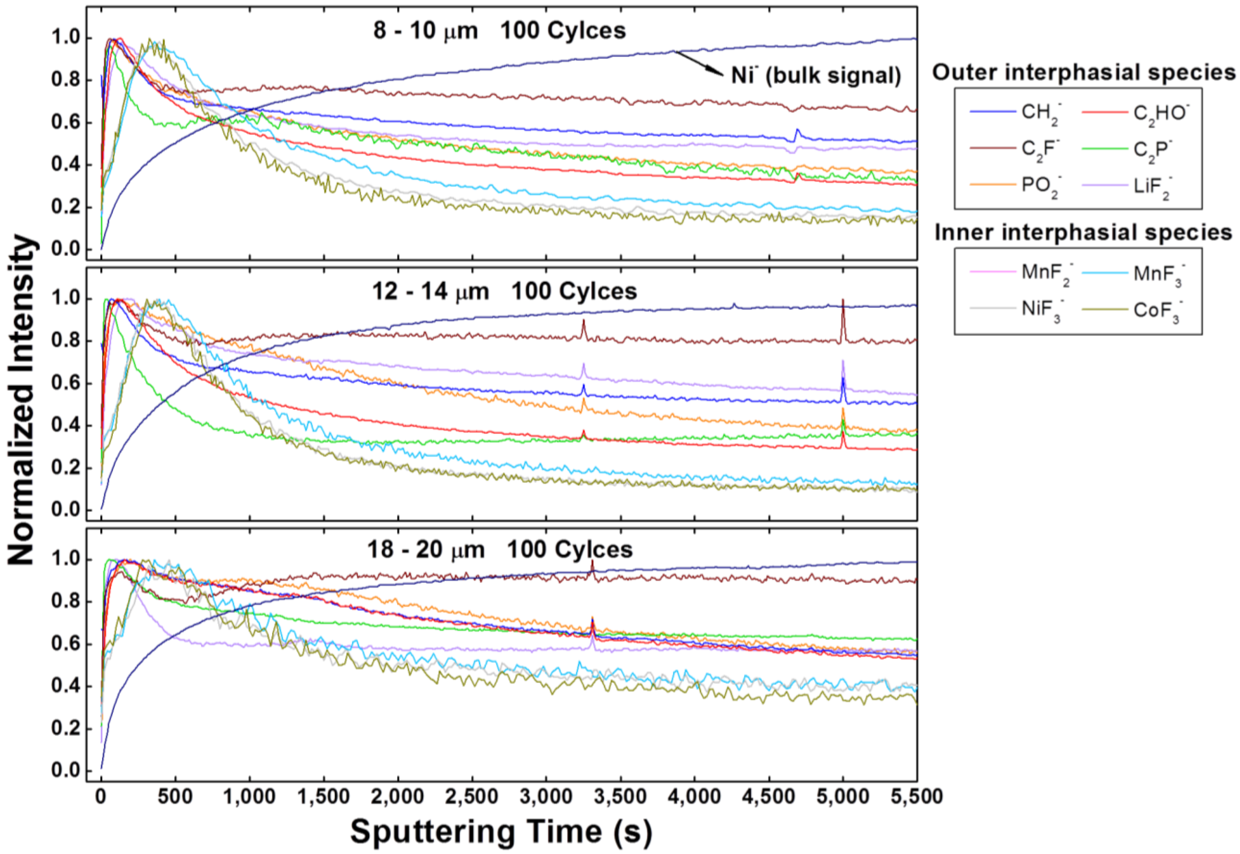

Supplementary Figure 24: Normalized (to maximum) TOF-SIMS depth profiles of outer and inner interphasial species representative secondary ion fragments for the 8-10 μm, 12-14 μm and 18-20 μm composite electrodes after 100 cycles.

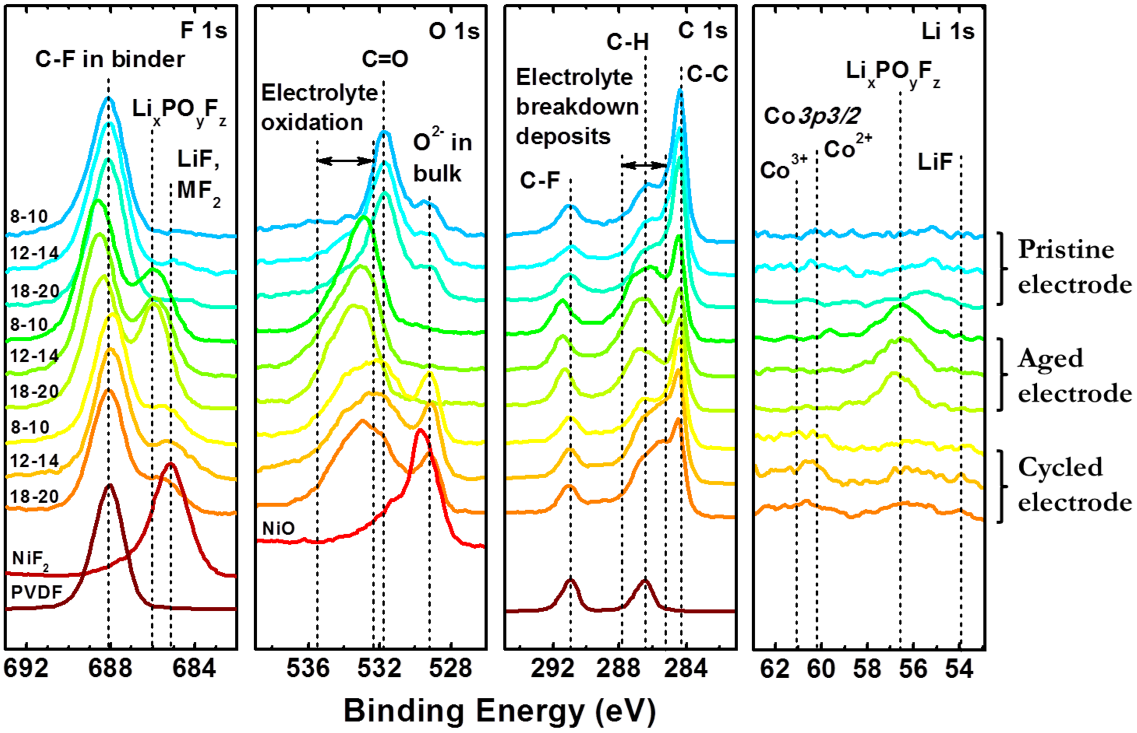

Supplementary Figure 25: High-resolution XPS spectra of the $LiNi_{0.7}Co_{0.15}Mn_{0.15}O_2$ samples with different secondary particle sizes and cycling conditions. Spectra are grouped by pristine, aged (without the electrochemical implementation), and cycled from the top to bottom. Spectroscopic data of NiO, NiF_2, and binder in the composite electrodes (PVDF) are also present for comparison. Particle size is indicated in the figure in each group. From left to right: F 1s, O 1s, C 1s, and Li 1s (also with Co 3p).

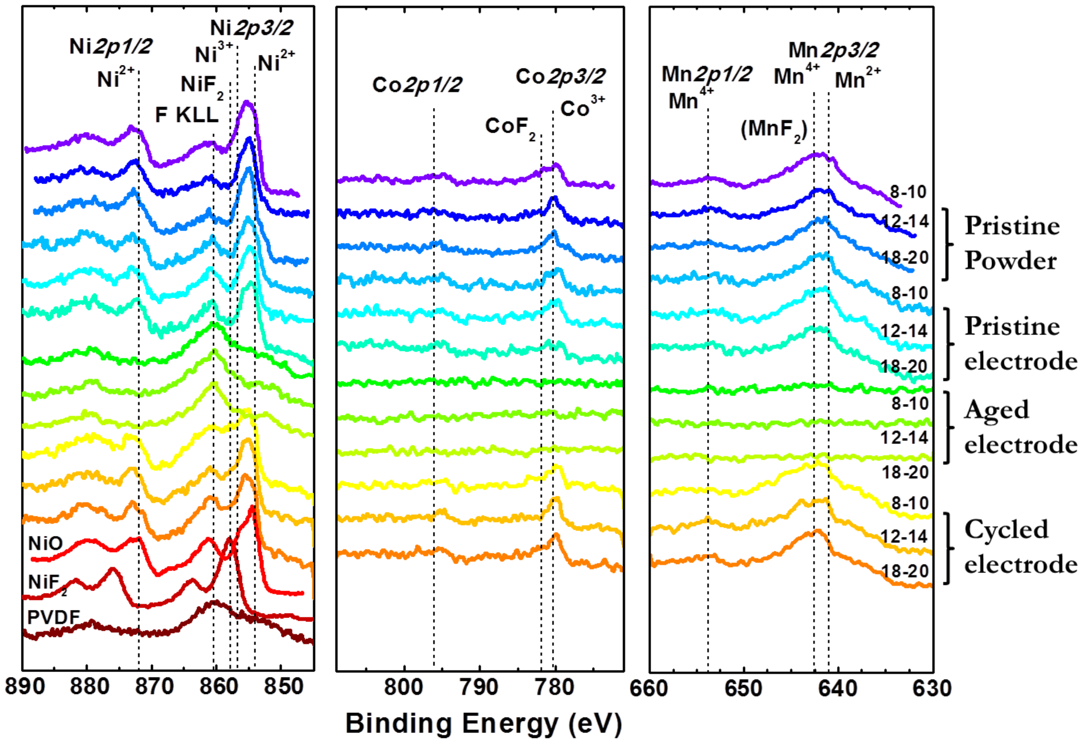

Supplementary Figure 26: (Continuing from Supplementary Fig. 33) from left to right: Ni 2p, Co 2p, and Mn 2p.

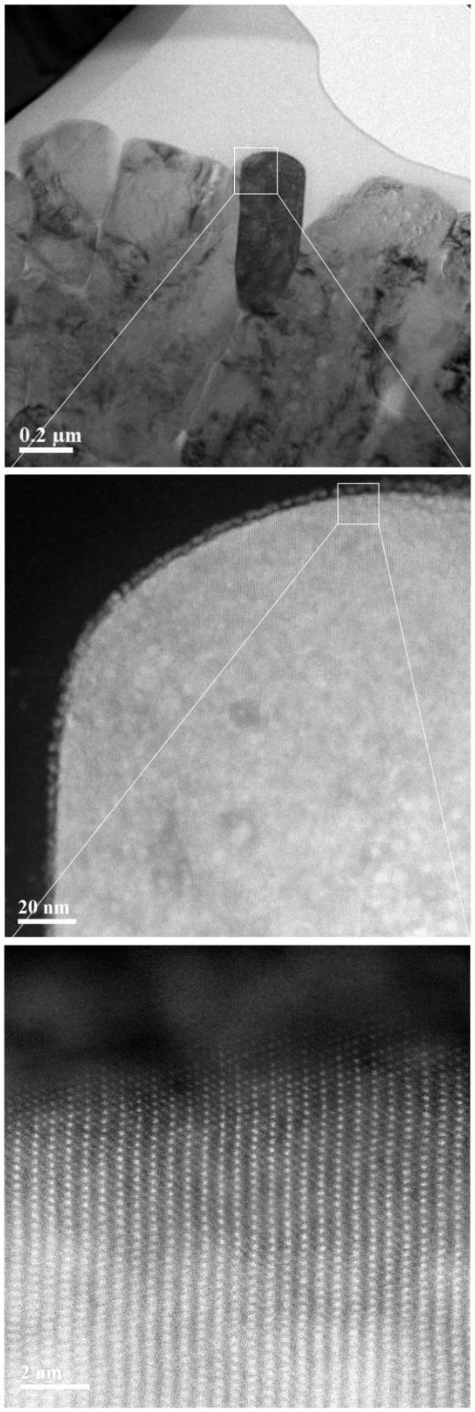

Supplementary Figure 27: HAADF-STEM images of pristine 8–10 µm $LiNi_{0.7}Co_{0.15}Mn_{0.15}O_2$ material. The images show that the primary particle surface of the pristine sample is free of the electrochemically generated rock-salt phase (NiO); only a thin layer (~ 2 nm) of the cation-mixing phase is present at the surface.

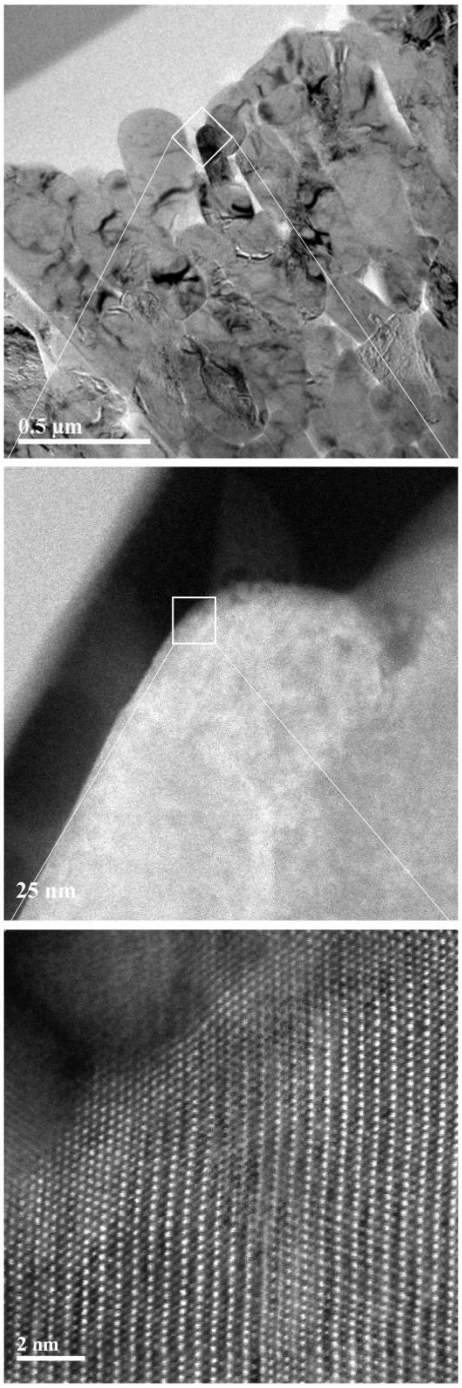

Supplementary Figure 28: HAADF-STEM images of pristine 18–20 µm $LiNi_{0.7}Co_{0.15}Mn_{0.15}O_2$ material. Likewise, these images indicate that the primary particle surface of the pristine sample is free of the electrochemically generated rock-salt phase (NiO); only a thin layer (~ 2 nm) of the cation-mixing phase is present at the surface.

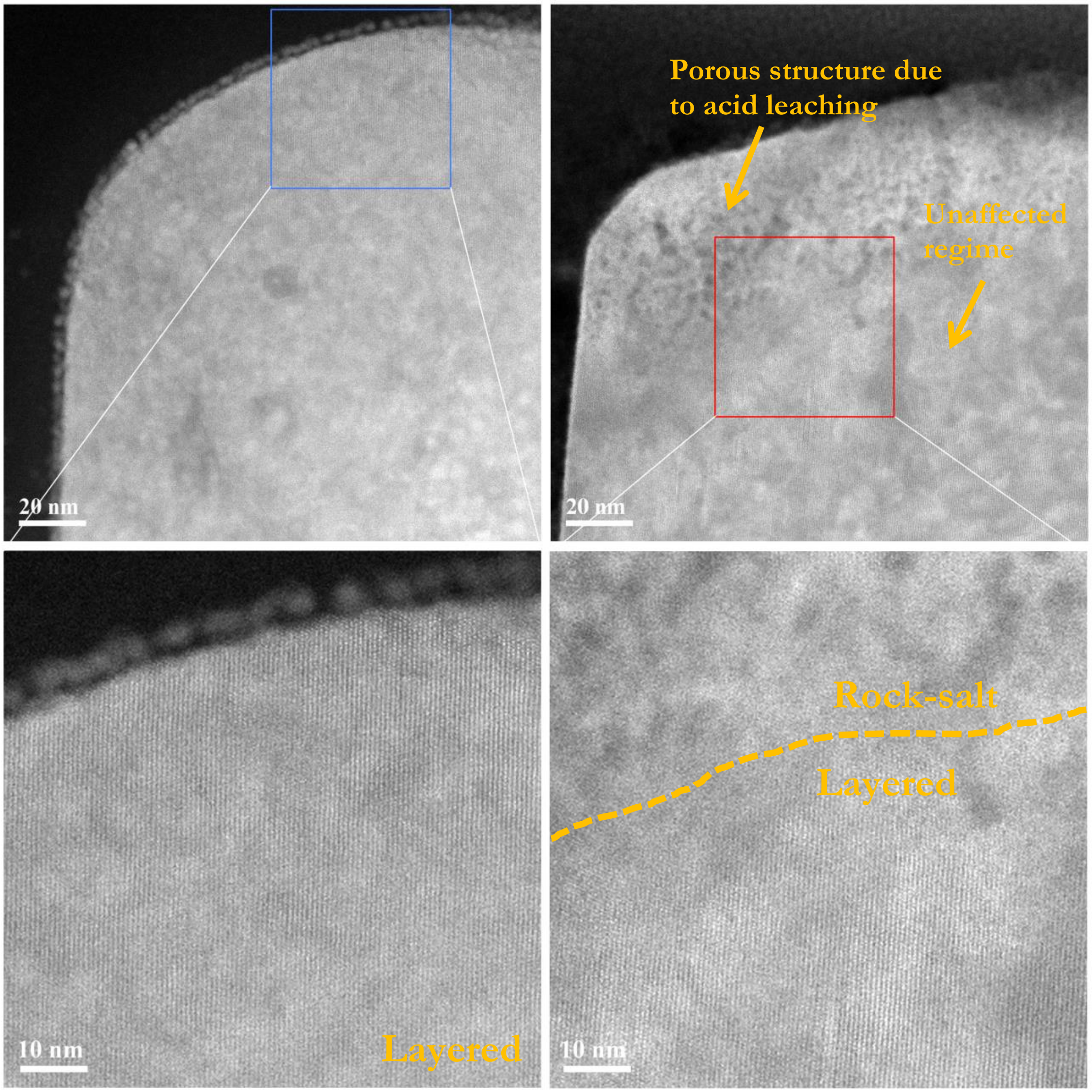

Supplementary Figure 29: High-resolution transmission electron microscopy images of the pristine (left) and cycled (right) $LiNi_{0.7}Co_{0.15}Mn_{0.15}O_2$ primary particle surface. Clearly, after 100 cycles at room temperature, the rock-salt phase becomes thick (more than 50 nm) along the lithium diffusion channels in the layered host lattice. It can also been seen that the rock-salt phase is porous, compared to the pristine material.

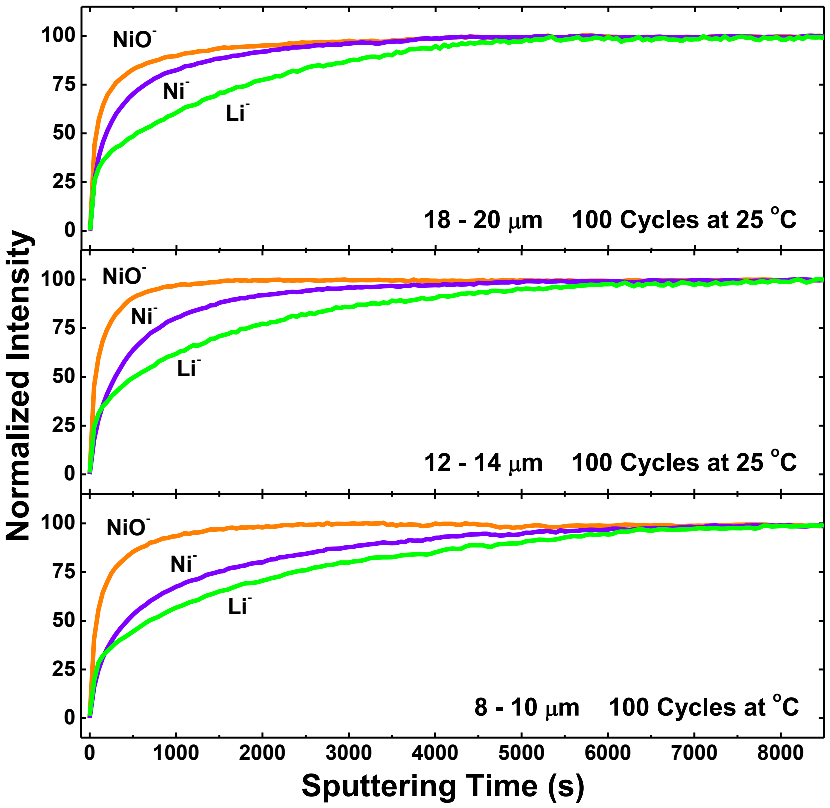

Supplementary Figure 30: Normalized (to maximum) TOF-SIMS depth profiles of two fragments of interest, NiO^- and Ni^-, whose ratio represents the rock-salt phase, for the 18-20 μm, 12-14 μm and 8-10 μm composite electrodes after 100 cycles, showing the rock-salt phase increases (that is, the increasing separation between the NiO^- and Ni^- normalized signals at the surface) with decreasing particle size.

$LiNi_{0.7}Co_{0.15}Mn_{0.15}O_2$	8–10 μm		12–14 μm		18–20 μm	
Cell parameters	*a*-axis (Å)	*c*-axis (Å)	*a*-axis (Å)	*c*-axis (Å)	*a*-axis (Å)	*c*-axis (Å)
	2.8795	14.2558	2.8779	14.2484	2.8785	14.2495
Cation Disordering	3.5%		2.9%		3.4%	
BET surface area	0.60 m^2 g^{-1}		0.50 m^2 g^{-1}		0.43 m^2 g^{-1}	

Supplementary Table 1: Calculated lattice parameters and cation disordering (Li/Ni mixing) based on the Rietveld refinement in Supplementary Fig. 9 for $LiNi_{0.7}Co_{0.15}Mn_{0.15}O_2$ powder of different secondary particle size. The Li/Ni mixing arises owing the similar ionic radii of Li^+ and Ni^{2+}. Also shown is the Brunauer-Emmett-Teller surface area of three samples. It is expected that the sample with larger secondary particles exhibit a relatively smaller surface area.

**Component**	**SI of interest**	**Notes**
$ROCO_2Li$	CH_2^-, C_2HO^- $C_3H_2^-$, CH_3O^-	Semicarbonates mostly occur in carbonates-containing electrolytes, such as ethylene, diethyl, or dimethyl carbonates.
Polycarbonates	CH_2^-, C_2HO^- $C_3H_2^-$, CH_3O^-	One possible route for the formation of these compounds are believed to be the (oxidative) polymerization of cyclic carbonates in electrolytes.
**RCF_x, RCO_xF_y**	CH_2^-, C_2HO^-, C_2F^-, C_3OF^-, $C_3O_2F^-$, *etc.*	Fluorinated organic species are usually generated when carbon blacks or carbonate solvents in electrolyte solutions react with HF.
LiF, $Li_xPO_yF_z$	C_2P^-, $^7LiF_2^-$, PO_2^-, POF_2^-	Commonly found in electrolyte solutions that employ fluorinated salts such as $LiPF_6$ and $LiBF_4$. Despite being a salt decomposition product, LiF can also be formed from reactions between semicarbonates/cathode-materials and HF.
Li_2O, Li_2CO_3	$^7LiO^-$, $^7LiO_2^-$	Readily formed in native surface film during material synthesis/storage, characteristic on transition metal oxides. Li_2CO_3 is generated when Li_2O reacts with moisture and CO_2 in ambient environment.
**MF_x (M = metal)**	MnF_2^-, $^{58}NiF_3^-$, CoF_3, MnF_3^-, $^7LiF_2^-$	Typical dissolution products of active cathode materials in electrolytes, especially with the presence of acidic impurities (*e.g.*, hydrolysis of PF_6^-).
NiO	$^{58}Ni^-$, $^{58}NiO^-$	It is present on the surface of nickel-rich layered oxides upon electrochemical implementation. At the highly delithiated stage, the tetravalent Ni ions tend to migrate to the neighboring vacant Li sites during which the original layered $R\bar{3}m$ structural configuration transforms into the rock-salt $Fm\bar{3}m$ phase.

Supplementary Table 2: Common components present in the interphases spontaneously and electrochemically formed on layered high-energy cathode oxides upon electrochemical operation. The irreversibly formed rock-salt phase (NiO) is also included. In this study, we particularly focus on the fluorinated organic species (major components in CEI) and metal fluorides (major active mass dissolution products) in the cathode interphases.

Cycle number	#	CH_2^-	C_2HO^-	C_2F^-	C_3OF^-
**3**	1	85	100	83	76
	2	80	106	71	86
	3	91	122	80	92
**Mean: 89**	4	84	104	73	85
**σ: 5.9**	Mean	85	108	77	85
	σ_i	3.9	8.4	4.9	5.7
**20**	1	92	132	89	124
	2	78	114	80	116
	3	99	104	75	135
**Mean: 101**	4	95	116	91	106
**σ: 11.1**	5	68	102	101	100
	Mean	86	114	87	116
	σ_i	11.6	10.7	9.0	12.5
**100**	1	84	132	87	101
	2	90	139	76	112
	3	104	120	93	93
**Mean: 105**	4	111	114	89	108
**σ: 9.8**	5	116	114	94	122
	Mean	101	124	88	107
	σ_i	12.2	10.0	6.4	9.8

Supplementary Table 3: The CEI formation depth was calculated from averaging the peak positions of the depth profiles for four representative fragments of interest (CH_2^-, C_2HO^-, C_2F^-, and C_3OF^-) for interphasial species on cathode particle surface migrated from carbon black prior to electrochemical operation. Data were collected at multiple locations on the composite electrodes (mostly 5) and this table summarizes those for the 8–10 μm $LiNi_{0.7}Co_{0.15}Mn_{0.15}O_2$ electrodes (with ROI-1 applied). The standard deviation is obtained by:

$$\sigma = \sqrt{\frac{1}{4}\sum_{i=1}^{4}(\sigma_i)^2}$$

Subsequently, all obtained values are doubled to yield a reasonable estimate of the thickness of the CEI. For example, for the 8–10 μm $LiNi_{0.7}Co_{0.15}Mn_{0.15}O_2$ after 20 cycles, the CEI formation depth obtained here is ~ 202 ± 22 seconds (which translates to ~ 5.5 – 6.5 nm).

Cycle number	#	CH_2^-	C_2HO^-	C_2F^-	C_3OF^-
**3**<br><br>**Mean: 104**<br>**σ: 6.7**	1<br>2<br>3	116<br>129<br>105	112<br>98<br>112	86<br>94<br>97	100<br>102<br>92
	Mean	117	107	92	98
	σ_i	9.8	6.6	4.6	4.3
**20**<br><br>**Mean: 116**<br>**σ: 10.1**	1<br>2<br>3<br>4<br>5	120<br>99<br>107<br>136<br>141	121<br>120<br>125<br>132<br>145	93<br>99<br>98<br>109<br>97	112<br>114<br>125<br>116<br>110
	Mean	121	129	99	115
	σ_i	16.2	9.2	5.3	5.2
**100**<br><br>**Mean: 129**<br>**σ: 12.8**	1<br>2<br>3<br>4<br>5	169<br>152<br>125<br>157<br>131	139<br>145<br>140<br>116<br>143	121<br>118<br>116<br>96<br>114	137<br>112<br>116<br>102<br>139
	Mean	147	137	113	121
	σ_i	16.4	10.5	8.8	14.5

Supplementary Table 4: (Continuing from Supplementary Table 3) this table displays data for the $LiNi_{0.7}Co_{0.15}Mn_{0.15}O_2$ electrodes of 12–14 μm in particle size (ROI-1 applied).

Cycle number	#	CH_2^-	C_2HO^-	C_2F^-	C_3OF^-
**3**	1	116	96	89	90
	2	129	114	104	105
	3	108	127	96	109
**Mean: 104**	4	102	108	94	85
**σ: 9.5**	Mean	114	111	96	97
	σ_i	8.4	11.1	5.4	10.0
**20**	1	117	174	146	120
	2	100	145	116	148
	3	178	131	119	130
**Mean: 136**	4	120	136	121	131
**σ: 20**	5	142	188	105	146
	Mean	131	155	121	135
	σ_i	27	22	13.4	10.5
**100**	1	133	139	110	113
	2	118	168	123	110
	3	108	120	113	107
**Mean: 130**	4	148	211	121	142
**σ: 19**	5	132	140	127	123
	Mean	122	156	119	119
	σ_i	11.5	32	6.3	12.7

Supplementary Table 5: (Continuing from Supplementary Table 4) data for the 18–20 μm $LiNi_{0.7}Co_{0.15}Mn_{0.15}O_2$ cycled electrodes (with ROI-1) are shown in this table.

Cycle number	#	MnF_3^- + $^{58}NiF_3^-$ + CoF_3^-
**3**	1	59312
	2	91970
	3	94703
	4	57396
	**Mean**	**75845**
	**σ**	**17531**
**20**	1	186709
	2	149025
	3	206008
	4	164268
	5	155954
	**Mean**	**172393**
	**σ**	**21058**
**100**	1	332409
	2	248747
	3	269300
	4	177492
	5	140010
	**Mean**	**233592**
	**σ**	**68080**

Supplementary Table 6: The degree of active mass dissolution was estimated from combining the integrated yield of three representative fragments of interest (MnF_3^-, $^{58}NiF_3^-$, and CoF_3^-) during the TOF-SIMS measurement (sputtering time: 750 s; 2 scans per 10 s). Data were collected at multiple locations on the composite electrodes (mostly 5) and this table summarizes those for the 8–10 μm $LiNi_{0.7}Co_{0.15}Mn_{0.15}O_2$ electrodes (with ROI-1 applied and normalized by ROI coverage). The unit is 'total counts'.

Cycle number	#	$MnF_3^- + {}^{58}NiF_3^- + CoF_3^-$
**3**	1	46943
	2	44664
	3	27272
	**Mean**	**39627**
	**σ**	**8785**
**20**	1	110811
	2	88270
	3	75688
	4	64784
	5	59745
	**Mean**	**79860**
	**σ**	**18319**
**100**	1	160326
	2	162704
	3	282298
	4	159016
	5	187198
	**Mean**	**190309**
	**σ**	**47142**

Supplementary Table 7: (Continuing from Supplementary Table 6) this table displays data for the $LiNi_{0.7}Co_{0.15}Mn_{0.15}O_2$ electrodes of 12–14 μm in particle size (ROI-1 applied and normalized by ROI coverage).

Cycle number	#	MnF_3^- + $^{58}NiF_3^-$ + CoF_3^-
**3**	1	49365
	2	51211
	3	66053
	4	29482
	**Mean**	**49028**
	**σ**	**13037**
**20**	1	41134
	2	74883
	3	44295
	4	49497
	5	49940
	**Mean**	**51950**
	**σ**	**11929**
**100**	1	110625
	2	127592
	3	85339
	4	93075
	5	150084
	**Mean**	**113343**
	**σ**	**23475**

Supplementary Table 8: (Continuing from Supplementary Table 7) data for the 18–20 μm $LiNi_{0.7}Co_{0.15}Mn_{0.15}O_2$ cycled electrodes (with ROI-1 and normalized by ROI coverage) are shown in this table.
